# *Endozoicomonas* Are Specific, Facultative Symbionts of Sea Squirts

**DOI:** 10.3389/fmicb.2016.01042

**Published:** 2016-07-12

**Authors:** Lars Schreiber, Kasper U. Kjeldsen, Peter Funch, Jeppe Jensen, Matthias Obst, Susanna López-Legentil, Andreas Schramm

**Affiliations:** ^1^Department of Bioscience, Center for Geomicrobiology and Section for Microbiology, Aarhus UniversityAarhus, Denmark; ^2^Section of Genetics, Ecology and Evolution, Department of Bioscience, Aarhus UniversityAarhus, Denmark; ^3^Department of Marine Sciences, University of GothenburgGothenburg, Sweden; ^4^Department of Biology and Marine Biology, Center for Marine Science, University of North Carolina WilmingtonWilmington NC, USA

**Keywords:** tunicates, sea squirts, ascidians, *Endozoicomonas*, symbiosis, mucin, marine

## Abstract

Ascidians are marine filter feeders and harbor diverse microbiota that can exhibit a high degree of host-specificity. Pharyngeal samples of Scandinavian and Mediterranean ascidians were screened for consistently associated bacteria by culture-dependent and -independent approaches. Representatives of the *Endozoicomonas* (Gammaproteobacteria, Hahellaceae) clade were detected in the ascidian species *Ascidiella aspersa, Ascidiella scabra, Botryllus schlosseri, Ciona intestinalis, Styela clava*, and multiple *Ascidia*/*Ascidiella* spp. In total, *Endozoicomonas* was detected in more than half of all specimens screened, and in 25–100% of the specimens for each species. The retrieved *Endozoicomonas* 16S rRNA gene sequences formed an ascidian-specific subclade, whose members were detected by fluorescence *in situ* hybridization (FISH) as extracellular microcolonies in the pharynx. Two strains of the ascidian-specific *Endozoicomonas* subclade were isolated in pure culture and characterized. Both strains are chemoorganoheterotrophs and grow on mucin (a mucus glycoprotein). The strains tested negative for cytotoxic or antibacterial activity. Based on these observations, we propose ascidian-associated *Endozoicomonas* to be commensals, living off the mucus continuously secreted into the pharynx. Members of the ascidian-specific *Endozoicomonas* subclade were also detected in seawater from the Scandinavian sampling site, which suggests acquisition of the symbionts by horizontal transmission. The combined results indicate a host-specific, yet facultative symbiosis between ascidians and *Endozoicomonas*.

## Introduction

Several species of ascidians (Tunicata, Ascidiacea), commonly referred to as sea squirts, have been shown to produce bioactive secondary metabolites (Faulkner, 2002), which are hypothesized to protect from biofouling and predation (e.g., Degnan et al., 1989; Paul et al., 1990; Fu et al., 1998; Vervoort et al., 1998; López-Legentil et al., 2006). At least in the case of the ascidian *Lissoclinum patella*, it was shown that these bioactive compounds are in fact produced by its bacterial symbiont *Prochloron didemni* (Lewin, 1981; Schmidt et al., 2005). The detection of defensive microbial symbionts in other marine invertebrates, such as corals, sponges and bryozoans (Faulkner, 2002), as well as the potential to use the produced bioactive compounds as anticancer drugs (Simmons et al., 2005; Erwin et al., 2010) has sparked an increased interest for bacterial symbionts of ascidians. The known diversity of ascidian-associated bacteria comprises members of several phyla including Actinobacteria, Bacteroidetes, Cyanobacteria, and Firmicutes, as well as Alpha-, Gamma-, and Delta-proteobacteria (Schuett et al., 2005; Martínez-García et al., 2007, 2010; Tait et al., 2007; Erwin et al., 2012, 2014; Dishaw et al., 2014; Tianero et al., 2014; López-Legentil et al., 2015b, 2016).

Ascidians are filter feeders and can pump tens of liters of water through their bodies per day (Petersen and Riisgård, 1992). Water enters through the buccal siphon, passes through a perforated and ciliated pharynx covered with a mucus-net that is produced continuously and traps minute food particles down to the micrometer range, before it leaves through the atrial siphon (MacGinitie, 1939; Bone et al., 2003). The mucus net together with entrapped food particles is eventually transported to the ascidian gut for digestion (MacGinitie, 1939). To our knowledge, microbiomes of ascidian pharynges have so far only been explored by a single study (Moss et al., 2003), while most other studies focused on the tunic (Schuett et al., 2005; Martínez-García et al., 2007, 2010; Tait et al., 2007; Erwin et al., 2012, 2014; López-Legentil et al., 2015a,b), the gonads (Tait et al., 2007; Erwin et al., 2012), or the gut (Dishaw et al., 2014). However, in contrast to the tunic, which is constantly exposed to the external environment, or the gonads and gut, which are located relatively secluded within the visceral cavity, the pharynx appears to be a well-suited habitat for bacterial symbionts: (i) it offers protection by being located within the ascidian body, and (ii) the large flow of seawater through the pharynx offers a constant supply of nutrients.

We aimed to explore the bacterial diversity associated with the pharynx tissue of ascidians in order to identify potential symbiotic lineages. The term “symbiosis” is here used in its broadest sense as the “living together of unlike organisms” (*sensu* De Bary, 1879) and thus encompasses mutualism (the fitness of both organisms is improved), commensalism (one organism's fitness is improved with no negative effect on the fitness of the other), and parasitism (one organism's fitness is improved while the fitness of the other is negatively affected). The frequent recovery of sequences affiliated with the *Endozoicomonas* clade (Gammaproteobacteria, Hahellaceae) indicated a symbiosis between *Endozoicomonas* and ascidians, and consequently led us to specifically focus on these bacteria. The aims of this study were thus (i) to test if *Endozoicomonas* are generally and specifically associated with ascidians, and (ii) to investigate the interaction between *Endozoicomonas* and ascidians. These aims were pursued using Scandinavian and Mediterranean ascidian specimens and a combination of culture-dependent and -independent approaches.

## Materials and methods

### Sample collection and identification

Specimens of the ascidian genera *Ascidia* and *Ascidiella*, as well as of the species *Botryllus schlosseri, Ciona intestinalis*, and *Styela clava* were collected from Gullmarsfjorden, Sweden, and from Limfjorden, Denmark (Figure S1), between September 2009 and October 2011. Three *A. aspersa* specimens were collected along the Mediterranean coast of Spain (Figure S1) in January/February 2013. Detailed metadata of all sampled specimens are listed in Table S1. Ascidian species were identified using both morphological (Lützen, 1967; Turon, 1987) and molecular approaches (see below).

Specimens BS-1 to BS-6 of the colonial ascidian *B. schlosseri* (where zooids share a single tunic) were sampled as whole animals. Specimens AJ-1 to AJ-4 were too small for dissection and were also processed as whole animals. Whole animals were rinsed with sterile seawater before DNA extraction. All remaining ascidian specimens were dissected. Their pharynx tissues were removed and rinsed with sterile water or seawater to remove cross-contamination from seawater. Due to the small size of specimen AA-15, gut, and pharynx of this animal could not be separated and were sampled together. The sizes of the remaining dissected specimens made cross-contamination of the sampled pharynx tissues with other tissue types very unlikely. The DNA of whole animals or pharynx tissues, respectively, was extracted with the DNeasy Blood and Tissue Kit (Qiagen, Venlo, The Netherlands). This extracted DNA was the basis for ascidian species identification and for identifying ascidian-associated bacteria.

For taxonomic identification of specimens a fragment of the mitochondrial cytochrome c oxidase I gene (CO1) was amplified with primers LCO1490f and HCO2198R (Folmer et al., 1994). PCR products were re-amplified if the initial PCR yielded insufficient material for downstream analysis. PCR products of samples AV-10, AS-1, BS-1, CI-5, and SC-4 (Table S1) were purified with the Wizard® SV Gel and PCR Clean-Up System (Promega, Fitchberg, USA) and cloned using the pGEM®-T vector System (Promega) prior to Sanger sequencing. PCR products of all other samples were sequenced directly. All sequencing was performed by Macrogen Inc. (Seoul, South Korea).

CO1 sequences were aligned using ClustalW (Larkin et al., 2007) implemented in Geneious v5.6.3 (created by Biomatters; available from http://www.geneious.com) with the following parameters: gap opening cost 24 and gap extension cost 12. Final alignment positions of CO1 gene sequences were 589 bp for *C. intestinalis* and 586 bp for all other ascidians. Neighbour-joining (NJ) analysis was conducted in MEGA 5 (Tamura et al., 2011) using the Jukes-Cantor model of nucleotide substitution and 10,000 bootstrap replicates (Felsenstein, 1985). The software JModelTest2 (Guindon and Gascuel, 2003; Darriba et al., 2012) was used to select the best model of DNA substitution for maximum likelihood (ML) analysis according to the Akaike information criterion (AIC). The transitional model TIM3+I+G with substitution rates varying among sites according to an invariant gamma distribution was subsequently selected. Maximum likelihood analysis was conducted using the phangorn package for R (Schliep, 2011) using 1000 bootstrap replicates.

### PCR-based survey of bacteria associated with the pharynx tissue of ascidians

Bacterial 16S rRNA genes were amplified with primer sets 26F/1492R (Hicks et al., 1992; Muyzer et al., 1995; specimens AA-1 to AA-4, AA-12, AM-1, AM-2, AV-1, AV-12, AS-5, BS-2, and CI-2) and GM1F/Bac1075R (Muyzer et al., 1993; Ohkuma and Kudo, 1998; specimens AA-14 to AA-16). HotStar Taq Master Mix (Qiagen) and the following PCR conditions were used for primer set 26f/1492R: initial denaturing at 95°C for 15 min; 32 cycles at 92°C for 0.5 min, at the annealing temperature for 1 min, and 72°C for 1.5 min; final elongation at 72°C for 10 min. The annealing temperature was optimized for each sample using separate PCR reactions and varied between 52 and 57°C. For primer set GM1F/Bac1075R, Taq DNA Polymerase Master Mix RED (Ampliqon, Odense, Denmark) and the following PCR conditions were used: initial denaturing at 95°C for 5 min; 27 cycles at 93°C for 45 s, 57°C for 45 s, and 72°C for 1 min; final elongation at 72°C for 10 min. PCR products were cloned using the pGEM®-T vector System (Promega) and subsequently sequenced from one direction by Macrogen Inc. This yielded partial 16S rRNA gene sequences between 257 and 1178 bp in length. *Endozoicomonas* sequences of specimens AA-2, AA-3, AM-2, and AV-1 were sequenced to full length and assembled using Sequencher version 5.0.1 (Gene Codes, Ann Arbor, USA). Sequences were taxonomically classified with the ARB software package (Ludwig et al., 2004) based on the SILVA database, Release Ref NR 104 (Quast et al., 2013).

### Culture-based survey

Pharynx tissue of *A. aspersa* (AS-1), *A. scabra* (AS-2), *Ascidia* sp. (AM-5, AV-11), and *Ascidiella* sp. (AV-10) specimens were dissected and washed with filter-sterilized (pore size: 0.2 μm) seawater. Tissue samples were added to 50 μL sterile seawater and homogenized in 1.5 mL tubes using sterile polypropylene pestles. The homogenate was diluted 1:14 with sterile seawater and 80 μL aliquots of this suspension were spread on full and half-strength Difco marine agar plates (BD, Franklin Lakes, USA). Plates were incubated in the dark at either 4, 10, 15, or 22°C for 1 week and regularly checked for growth. Colonies with unique morphologies were purified by repeated streaking. Isolated strains were preserved at −80°C in marine broth supplemented with glycerol (30% final concentration).

Strains were identified by 16S rRNA gene sequencing: Single colonies were suspended in 100 μL PCR-grade H_2_O. Of this suspension, 1 μL was used as template for PCR with primers GM3 and GM4 (Muyzer et al., 1995). HotStar Taq Master Mix (Qiagen) and the following conditions were used for PCR amplification: initial denaturing at 95°C for 5 min; 36 cycles at 95°C for 1 min, 42°C for 1 min, and 72°C for 3 min; final elongation at 72°C for 10 min. PCR products were directly sequenced (Macrogen Inc.) using either the internal primer 341F (strains from specimens AM-5, AS-1, and AV-10)(Muyzer et al., 1993) or the primer set GM3/GM4 (strains from specimen AV-11). PCR products of two obtained *Endozoicomonas* strains (AVMART05 and KASP37) were cloned and sequenced using the vector primers M13F and M13R. Sequences were assembled using Sequencher version 5.0.1 (Gene Codes). Sequences were trimmed using the online SINA aligner of SILVA (Quast et al., 2013), which removed all bases at either sequence end that could not be aligned. Final 16S rRNA gene fragment lengths were: 616–936 bp for strains originating from specimens AM-5, AS-1, and AV-10; 948–1426 bp for strains originating from specimen AV-11; and 1501–1521 for strains AVMART05 and KASP37. Trimmed sequences were classified based on the best BLASTn hit (Camacho et al., 2009) against NCBI's database of 16S rRNA genes of described microbial species (release December 4th, 2015).

### Phylogenetic analysis of the *Endozoicomonas* clade

Reference 16S rRNA gene sequences affiliated with the *Endozoicomonas*-containing Hahellaceae family and with a sequence length of ≥1400 bp were retrieved from the SILVA SSU database release 123 (Quast et al., 2013). Nearly full-length *Endozoicomonas* 16S rRNA gene sequences generated in this study were aligned using SINA (Pruesse et al., 2012). Members of the *Endozoicomonas* clade can harbor multiple divergent paralogs of the 16S rRNA gene (this study; Figure S2). Phylogenetic analysis was restricted to the dominant paralog present in public databases, i.e., the paralog-1-type (Figure S2), which represented 827 out of the total 1216 *Endozoicomonas* sequences inspected in this study. Together with *Hahellaceae* sequences clustering outside the *Endozoicomonas* clade, the analyzed dataset comprised a total of 852 sequences. Sequences were aligned *de novo* using MAFFT-qinsi version 7.221, which also considers the secondary structure of RNA (Katoh et al., 2005). The final alignment contained 1659 sites. Phylogenies were reconstructed using ML, maximum parsimony (MP), and Bayesian inference (BI) approaches. ML analysis was performed as implemented in RAxML version 7.4.2 (Stamatakis et al., 2008) using the General Time Reversible (GTR; Tavaré, 1986) model of nucleotide substitution under the Γ model of rate heterogeneity (Yang, 1994, 1996). Maximum parsimony analysis was performed using the PHYLIP software package (version 3.69; Felsenstein, 2005). Node stability of ML and MP phylogenies was evaluated by 1000 bootstrap replicates. Bayesian inference-based analysis was performed using MrBayes 3.2.5 (Ronquist and Huelsenbeck, 2003). MrBayes was run for 2 million generations and trees were sampled every 1000 generations after a burn-in of 25%. Resulting tree topologies were compared using the relative Robinson-Foulds metric (Robinson and Foulds, 1981) as implemented in RAxML. A strict consensus tree summarizing ML, MP, and BI trees was calculated using the *consense* tool of PHYLIP, and annotated and visualized using the ARB software package (Ludwig et al., 2004). Obtained partial *Endozoicomonas* sequences were tested for affiliation with the ascidian-specific subclade based on the calculated BI phylogeny and using the Naïve Bayesian method (Wang et al., 2007) as implemented in the *classify.seqs* command of Mothur version 1.25 (Schloss et al., 2009). For classification, 1000 iterations and a confidence score threshold of 80% were used. Sequences of the paralog-2-type (Figure S2) were removed prior to analysis.

### *Endozoicomonas*-specific screening

Ascidians and four water samples from the Gullmarsfjord sampling site (Table S1) were screened for bacteria affiliating with the *Endozoicomonas* clade by semi-specific PCR using primers GM1F (Muyzer et al., 1993) and ENDO-1240R (AAC CGT CTG TAT GCA CCA; for further details see section below on *Endozoicomonas*-specific probe design).

Water samples (0.5 L) were collected onto polycarbonate filters (pore size: 0.2 μm) and extracted using the PowerLyzer PowerSoil DNA Isolation Kit (MOBIO Laboratories Inc., Carlsbad, USA) prior to PCR. The following PCR conditions were used: initial denaturing at 95°C for 15 min; 30 cycles at 92°C for 30 s, 52°C for 1 min, and 72°C for 1.5 min; final elongation at 72°C for 10 min. For the majority of specimens and the water samples, PCR products were cloned and sequenced as described above. Instead of cloning, PCR products of specimens AM-4, AA-7, and AA-11 were separated by denaturing gel electrophoresis (DGGE) as follows: PCR products were re-amplified with the primer set 341F-GC/907R (Lane, 1991; Muyzer et al., 1993) using the following PCR conditions: initial denaturing at 95°C for 15 min; 30 cycles at 92°C for 30 s, 55°C for 1 min, and 72°C for 1.5 min; final elongation step at 72°C for 10 min. The resulting PCR products were separated by DGGE on polyacrylamide gels containing a gradient of 20–80% denaturant; 100% denaturant was 7 M urea and 40% (v/v) deionized formamide (BioRad, Hercules, USA). The gels were cast and run as described earlier (Nicolaisen and Ramsing, 2002), and stained in 1 × SYBR Gold (Molecular Probes, Leiden, Netherlands) solution. Resulting DGGE bands were excised for subsequent sequencing. All excised bands were re-amplified by PCR with the primer set 341F/907R, and subsequently sequenced directly by Macrogen Inc.

### *Endozoicomonas* distribution and prevalence data from previous publications

Pyrosequencing 16S rRNA gene sequence data of previous studies targeting the bacterial diversity of several ascidian species (Erwin et al., 2012, 2014; Tianero et al., 2014; López-Legentil et al., 2016) were re-analyzed to obtain additional data on the prevalence of *Endozoicomonas* in ascidians. Data was quality trimmed using the prinseq-lite.pl script (Schmieder and Edwards, 2011) and subsequently pre-screened using Mothur 1.36.1 (Schloss et al., 2009) in combination with sequences and taxonomy classification of the “All-Species Living Tree” Project (LTP) database release 119 (Yarza et al., 2008). Data of specimens with a significant proportion of Hahellaceae-affiliated sequences were cleaned of eukaryotic sequences and analyzed thoroughly using the SILVAngs pipeline (Quast et al., 2013). Overall, operational taxonomic units with an abundance of < 1% were classified as absent (Degnan and Ochman, 2012). Additionally, *Endozoicomonas* 16S rRNA gene sequences of DGGE data (FJ659121, FJ659156) and of PCR amplicon libraries (DQ884160, DQ884169, DQ884170) from Martínez-García et al. (2007, 2010), as well as data from Tait et al. (2007), and López-Legentil et al. (2015a) were identified based on their taxonomic classification in the SILVA SSU database release 123 (Quast et al., 2013).

### Design, evaluation, and optimization of probes specific for ascidian-associated *Endozoicomonas*

Two oligonucleotide probes targeting ascidian-derived *Endozoicomonas* 16S rRNA gene sequences were designed using the ARB probe tool (Ludwig et al., 2004): probe ENDO-580 (name according to Alm et al. (1996): S-^*^-Endo-0580-a-A-18; probe sequence [5′-3′]: CAA CTT AAG TAG CCG CCT), and probe ENDO-1240 (S-^*^-Endo-1240-a-A-18; AAC CGT CTG TAT GCA CCA). Specificity of the probes was tested *in silico* using TestProbe 3.0 and the SILVA database release 123, as implemented on the SILVA website (Quast et al., 2013). Besides *Endozoicomonas* 16S rRNA genes, probe ENDO-580 targets also a small fraction of sequences (0.16%) within the Legionellales. Probe ENDO-1240 also targets sequences within the Rhodospirillales (0.06%), Sphingomonadales (0.01%), Thiotrichales (0.06%), and Vibrionales (0.04%). Sensitivity of the probes was evaluated *in silico* using the ARB software package (Ludwig et al., 2004) based on the *Endozoicomonas* phylogeny reconstructed in this study. Probe ENDO-580 targets 99% of the sequences affiliated with the ascidian-specific *Endozoicomonas* subclade. Almost all other *Endozoicomonas* sequences have 1–3 mismatches to the probe sequence. Probe ENDO-1240 targets 91% of the sequences affiliated with the ascidian-specific *Endozoicomonas* subclade and 27% of the *Endozoicomonas* sequences outside this subclade. *In situ* specificity and optimal hybridization conditions of the probes were evaluated using an *Endozoicomonas* strain isolated in this study whose 16S rRNA gene matches both probes perfectly (strain AVMART05; Table S2). *Endozoicomonas elysicola* DSM 22380 and *Lewinella nigricans* DSM 23189 were used as one-mismatch-controls for ENDO-580 or ENDO-1240, respectively. Fluorescence *in situ* hybridization (FISH) with Cy3-labeled oligonucleotide probes (Biomers.net, Ulm/Donau, Germany) was performed as described earlier (Fuchs et al., 2007) with a series of formamide concentrations: 0%, and 10 to 55% (in 5% steps). When used with helper probes (Fuchs et al., 2000), hENDO-559 (S-^*^-Endo-0559-a-A-21; CAC GCT TTA CGC CCA GTA ATT) and hENDO-604 (S-^*^-Endo-0604-a-A-21; GGT TGA GCC CGG GGC TTT CAC), probe ENDO-580 showed good signal intensity and single mismatch discrimination in hybridizations with 35% formamide. Signal intensity of probe ENDO-1240 was good in hybridizations with up to 35% formamide. However, single mismatch discrimination was not achieved at these conditions.

### Fluorescence *in situ* hybridization

Dissected pharynx tissue of specimens AA-14 (*A. aspersa*), AS-5 (*A. scabra*), and AV-12 (*Ascidia* sp.) were washed in sterile-filtered seawater and fixed in 2% paraformaldehyde solution for 1 h at room temperature. Tissue sections were consecutively washed in PBS (130 mM NaCl, 10 mM Na_2_HPO_4_/NaH_2_PO_4_; pH 7.4) and MilliQ, dried onto Superfrost™ Plus microscope slides (ThermoFisher Scientific, Waltham, USA), dehydrated in an ethanol series (50%, 70%, 96%; 3 min each), and stored at −20°C. Prior to FISH, tissue sections were circled with a hydrophobic PAP-pen (Kisker Biotech, Steinfurt, Germany) to create “hybridization wells.” To saturate unspecific binding sites of the sticky pharynx tissue, the sections were pre-hybridized at 46°C with hybridization buffer containing 35% formamide (v/v), 0.1% bovine serum albumin, 200 μg mL^−1^ salmon sperm DNA (Sigma-Aldrich, St. Louis, USA), 1% blocking reagent (Roche, Basel, Switzerland), 0.9 M NaCl, 20 mM Tris-HCl (pH 8), and 10 mM sodium dodecyl sulfate. FITC-, CY3-, or CY5-labeled oligonucleotide probes (final concentration, 1 pmol μL^−1^; Biomers.net) were added after 2 h, and hybridization proceeded for 90–120 min at 46°C. Slides were washed for 15 min in 50 mL pre-heated standard washing buffer (Fuchs et al., 2007) at 48°C, rinsed with MilliQ water, counterstained with 4,6-diamidino-2-phenylindole (DAPI) and mounted in a 4:1 mix of Citifluor (Citifluor Ltd., London, UK) and Vecta Shield (Vector Laboratories Inc., Burlingame, USA). Hybridized samples were examined and imaged with an epifluorescence microscope (Axiovert 200M; Carl Zeiss, Jena, Germany). Additional micrographs were obtained by confocal laser scanning microscopy (LSM700; Carl Zeiss).

### Functional analysis of *Endozoicomonas* isolates

A diffusion assay and an agar overlay assay were used to test for antibacterial activity of the *Endozoicomonas* isolates against gram-negative (*Escherichia coli* K-12 JM109) and gram-positive (*Bacillus cereus* ATCC 10987 and *Staphylococcus epidermidis* DSM 20044) indicator strains. For the diffusion assay, the isolates were grown in 50 mL marine broth in 250 ml Erlenmeyer flasks at 28°C and 100 rpm for 14 days. Twice a week, 1 mL of the culture was sampled and centrifuged at 13,400 g for 5 min to obtain a cell-free supernatant. The supernatant was sterile-filtered (pore diameter 0.22 μm) and stored at 4°C until used. LB plates were inoculated with 100 μL of a 1:50 dilution of an overnight culture of the indicator strain using sterile glass beads. Sterile paper discs (5 mm diameter) were placed onto the agar. Then, 15 μL cell-free supernatant, marine broth as negative control, or ampicillin solution (100 μg mL^−1^) as a positive control were applied to the paper discs. The plates were incubated for 24 h at 37°C and subsequently checked for zones of inhibition around the paper discs. For the agar overlay assay, 5 μL of test strain stock culture was spotted onto a marine agar plate and incubated at 28°C until the culture was approx. 0.5–1 cm in diameter (2–3 days). The cultures were then overlaid with 10 mL of LB agar inoculated with a fresh culture of indicator strain (200 μL of fresh overnight culture in 20 mL of 40°C-warm LB agar). The plates were incubated for 24 h at 37°C and subsequently checked for zones of inhibition.

Hemolytic activity was used as a predictor for cytotoxic activity (Gandhi and Cherian, 2000). It was tested by overlaying 5% sheep blood agar plates with marine agar. Freshly grown liquid cultures of the test strains (5 μL) were transferred onto the marine agar phase. The hemolytic test plates were incubated at 28°C for 4 days and regularly checked for clearing zones around the colonies. A beta-hemolytic *Vibrio splendidus* strain isolated from *Ascidia* sp. (specimen AM-5) was used as a positive control.

DNase activity was tested by overlaying methyl-green (Sigma-Aldrich) containing DNase test agar (Sigma-Aldrich; Smith et al., 1969) with marine agar. The plates were inoculated by streaking the test strain, incubated at 28°C for 48 h, and subsequently checked for clearing zones around the cultures. *E. coli* strain DSM 498 was used as a DNase negative control. The ability to metabolize DNA was tested using IF-A indicator medium (BIOLOG GENIII system; Biolog, Hayward, USA) supplemented with 2% NaCl. The indicator medium was inoculated with the test strain and dispensed into a 96-well plate. Duplicate wells of each strain were supplemented with dNTP's (final concentrations: 99, 291, and 566 μM), salmon sperm DNA (20, 58, and 113 mg L^−1^), or marine broth (0.09x and 0.18x). Plates were incubated for 7 days at 28°C and subsequently checked for purple coloration as indication that the provided substrate had been metabolized.

Mucin as a growth-supporting substrate of ascidian-derived *Endozoicomonas* isolates and *E. elysicola* DSM 22380 was tested with an agar-plate-based assay. Marine mucin agar was prepared containing the following (L^−1^): mucin from porcine stomach (Type II, Sigma-Aldrich; 10 g), ferric citrate (0.1 g), NaCl (19.45 g), MgCl_2_ (8.8 g), Na_2_SO_4_ (3.24 g), CaCl_2_ (1.8 g), KCl (0.55 g), NaHCO_3_ (0.16 g), KBr (80 mg), NH_4_NO_3_, (1.6 mg), Na_2_HPO_4_ (8 mg), agar (6.0 g). Prior to autoclaving, pH was adjusted to 7. After autoclaving, the mucin agar medium was cooled down to 45°C, before adding 1 mL vitamin (Widdel et al., 1983), 1 mL vitamin B12 (Widdel et al., 1983), and 2 mL trace metal SL-10 (Widdel and Bak, 1992) solutions per L. Liquid cultures of the tested strains were spotted on the mucin agar, incubated at 21°C for 48 h, and subsequently inspected for growth.

### Availability of sequences and isolates

Sequences obtained in this study were deposited at GenBank under the accession numbers KU647816-KU647849 (CO1 sequences of ascidian hosts), KT364255-KT364260 and KU647850-KU647930 (obtained isolates), and KU647931-KU648390 (culture-independent screenings). The obtained isolates are available upon request.

## Results and discussion

### Ascidian phylogeny

Phylogenetic analyses overall supported the morphology-based taxonomic identification of the ascidians. Thus all *A. aspersa* CO1 sequences formed a well-supported clade (bootstrap values >99% in all analyses, Figure S3). The CO1 sequences of *A. scabra* obtained in this study formed two separate but well-supported clades (bootstrap values >84%). The first clade grouped *A. scabra* sequences from the Mediterranean Sea with obtained sequences of juvenile specimens that could not be unambiguously identified by morphology (*Ascidiella* sp.). The second clade grouped all of the Swedish *A. scabra* sequences (bootstrap values >99%) and matched *A. scabra* sequences obtained from Atlantic individuals (Nishikawa et al., 2014). All the sequences obtained for the Scandinavian *Ascidia* sp. formed a single clade (bootstrap values >99%) within the Phlebobranchia. *C. intestinalis* sequences obtained in this study formed a well-supported clade with other *Ciona* sequences retrieved from GenBank (bootstrap values >99%), including *Ciona robusta* (formerly *C. intestinalis* type A; Brunetti et al., 2015). The confident separation of the *C. robusta* clade and our generated *Ciona* sequences indicates that the here studied animals were *C. intestinalis*. The last clade comprised all the Stolidobranchia samples analyzed here. All *S. clava* sequences (from this study and GenBank) formed a strongly supported clade (bootstrap values >99%) within the *Styela* spp. clade. Botryllid ascidians also formed a monophyletic clade, with all *B. schlosseri* sequences grouping together in a well-supported clade (bootstrap values >96%).

### Bacteria associated with pharynx tissue of ascidians

Rather than characterizing the bacterial diversity associated with pharynges of ascidians, the present study aimed at identifying potentially symbiotic bacteria, indicated by their general and specific association with ascidians, using culture-independent, and -dependent screenings. An initial screening of pharynx samples with general bacteria primers retrieved 16S rRNA gene sequences affiliating with genera of the Alphaproteobacteria (genera *Hoeflea* and *Roseobacter*), Gammaproteobacteria (*Alteromonas, Colwellia, Pseudoalteromonas, Pseudomonas, Vibrio, Neptuniibacter*, and *Endozoicomonas*), Deltaproteobacteria (*Halingium*), Epsilonproteobacteria (*Arcobacter*), and Fusobacteria (*Psychrilyobacter*) from more than one ascidian specimen (Table 1). Sequences affiliating with the *Endozoicomonas* genus were retrieved most frequently, and were detected in eight of twelve screened ascidian specimens.

**Table 1:** **Identity of ascidian-associated bacteria as detected by 16S rRNA gene amplification**.

**Taxonomic group**	***A. aspersa***			***A. scabra***				***Ascidia sp***.				***B. schlosseri***
	**AA-14**	**AA-15**	**AA-16**	**AA-2**	**AA-3**	**AA-12**	**AS-4**	**AA-1**	**AM-2**	**AV-1**	**AV-12**	**BS-2**
**ACIDOBACTERIA**												
Uncultured Holophagae	1	–	–	–	–	–	–	–	–	–	–	1
Actinobacteria	–	–	6 (1)	–	–	–	–	–	–	–	–	1
Chloroflexi	1	–	–	–	–	–	–	–	–	–	–	–
Cyanobacteria	–	–	–	–	–	–	–	–	–	–	–	2
**ALPHAPROTEOBACTERIA**												
*Hoeflea*	–	–	–	–	–	–	2 (1)	–	–	–	1	–
*Roseobacter*		–	–	–	–	1	7 (2)	–	–	–	–	–
Other	5 (4)	–	–	–	–	2	–	–	–	2	8	7
Betaproteobacteria	–	–	–	–	–	–	–	–	–	–	1	–
**GAMMAPROTEOBACTERIA**												
*Alteromononas*	3 (2)	–	–	–	–	5 (3)	1	–	–	–	5 (3)	–
***Colwellia***	–	–	–	–	–	**2 (2)**	–	–	–	–	**4 (2)**	–
***Pseudoalteromonas***	–	–	–	–	–	**2**	–	–	**1**	–	–	–
*Endozoicomonas*	**2 (1)**	**33 (6)**	–	**6 (5)**	**4**	**16 (4)**	**19 (13)**	–	**1**	**4 (3)**	**1**	**7 (2)**
*Neptuniibacter*	–	–	–	–	–	2	1	–	–	–	2	–
*Pseudomonas*	–	**1**	**1**	–	–	–	–	–	–	–	–	–
***Vibrio***	–	–	–	–	–	–	–	–	**2**	–	**1**	–
Other	–	2 (1)	3 (2)	–	–	2	–	–	–	–	2	5
**DELTAPROTEOBACTERIA**												
*Haliangium*	–	–	–	–	–	1	–	–	–	–	–	2
**EPSILONPROTEOBACTERIA**												
***Arcobacter***	–	–	–	–	–	**3 (2)**	**1**	–	–	–	**1**	–
Bacteroidetes	9 (4)	–	–	–	–	–	2	1	–	–	4	4
*Firmicutes/Bacilli*	–	–	3 (2)	–	–	–	–	–	–	–	–	–
**FUSOBACTERIA**												
*Psychrilyobacter*	–	–	–	–	–	–	–	3 (2)	–	–	2	–
Planctomycetes	–	–	–	–	–	–	1	–	–	–	-	3
**VERRUMICROBIA**												
*Roseibacillus*	1	–	5 (1)	–	–	–	–	–	1	–	–	–
Other	–	–	–	–	–	–	–	–	–	–	1	1
Clones analyzed	22	36	18	6	4	36	34	4	5	6	33	33

*Host specimens are shown with their identifiers. Genera also detected by culturing (Table 2) are shown in bold. Numbers represent the number of obtained clones for a given taxonomic group. Numbers in parentheses represent the number of obtained unique ribotypes (>0.5% sequence divergence) per group. −, no data*.

A complementary culture-based screening yielded several strains with similar taxonomic affiliations as those obtained with the culture-independent approach (Table 2). These isolates affiliated with (i) the gammaproteobacterial genera *Colwellia, Pseudoalteromonas, Vibrio*, and *Endozoicomonas*, and (ii) the epsilonproteobacterial genus *Arcobacter*. In addition, several isolates unique to the culturing approach were obtained. These affiliated mostly with the Gammaproteobacteria and included the genera *Aliivibrio, Acinetobacter, Microbulbifer, Moritella, Photobacterium, Shewanella*, and *Sinobacterium*. Additionally, two isolates affiliating with the genera *Flammeovirga* (Bacteroidetes) and *Bacillus* (Firmicutes), respectively, were uniquely obtained by culturing. The majority of the obtained isolates shared a 16S rRNA gene identity of >97% with described species (Table S2).

**Table 2:** **Identity of ascidian-associated bacteria as detected by culturing**.

**Phylogenetic affiliation**	***A. aspersa***	***A. scabra***	***Ascidia sp***.		***Ascidiella sp***.
	**AS-1**	**AS-2**	**AM-5**	**AV-11**	**AV-10**
**ALPHAPROTEOBACTERIA**					
*Tropicibacter*	**–**	**–**	**1**	**–**	**–**
**GAMMAPROTEOBACTERIA**					
*Microbulbifer*	3	–	–	–	1
***Colwellia***	**–**	**–**	**5**	**1**	**–**
***Pseudoalteromonas***	**–**	**–**	**–**	**3**	**–**
*Shewanella*	1	–	6	16	3
***Endozoicomonas***	**–**	**1**	**–**	**–**	**13**
*Photobacterium*	1	–	1	–	2
***Vibrio***	**–**	**–**	**8**	**7**	**–**
Other	–	–	6	–	1
**EPSILONPROTEOBACTERIA**					
***Arcobacter***	**1**	**–**	**1**	**–**	**–**
Bacteroidetes	–	–	1	–	–
*Firmicutes/Bacilli*	–	–	–	–	1
Unidentified	–	14	–	7	–
Total strains analyzed	6	15	29	34	21

*Numbers represent the number of obtained isolates for a given taxonomic group. Host specimens are shown with their identifiers. Genera also detected by 16S rRNA gene amplification (Table 1) are shown in bold. –, no data*.

**Figure 1:**
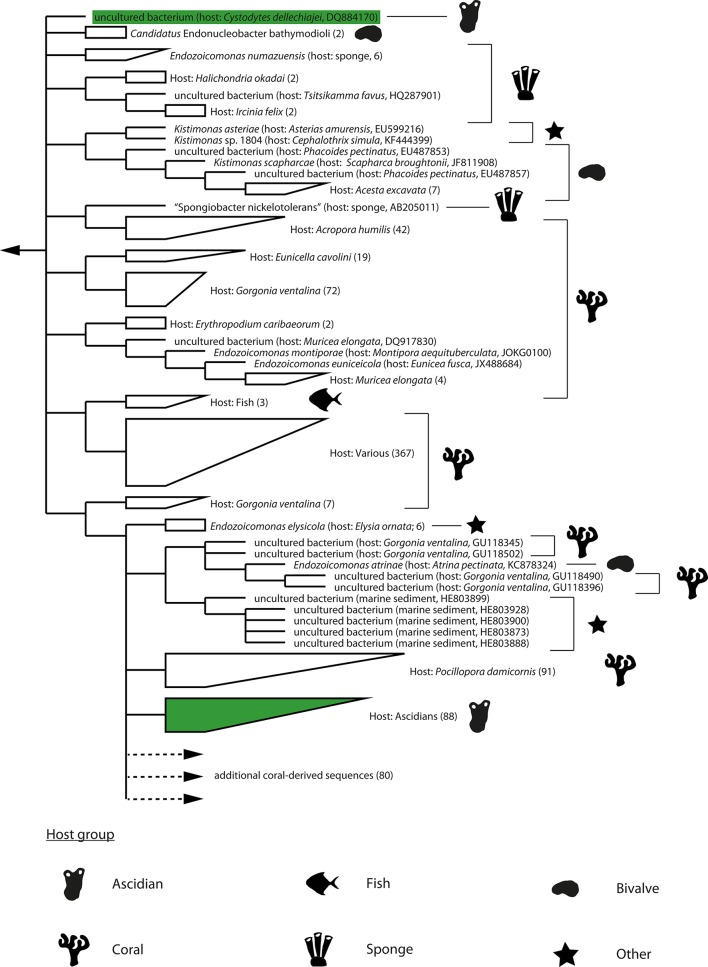
**Dendrogram of 16S rRNA gene sequences of the ***Endozoicomonas*** clade**. Strict consensus representation (i.e., the shown branching patterns and subclades were present in all source trees) of phylogenetic trees calculated by MP, ML, and BI analyses using only nearly full-length (≥1400 bp) 16S rRNA gene sequences. The dendrogram was rooted with sequences of the genera *Zooshikella* and *Hahella* (not shown). The dendrogram was truncated (indicated with dotted line and arrow) and does not show an additional 80 sequences of bacteria associated with coral hosts. Host species and accession numbers of single sequences are shown in brackets. For subclades, numbers of containing sequences are shown in brackets. Major host groups are indicated with silhouette symbols. Sequences of ascidian origin are additionally marked with green. Branch lengths do not represent phylogenetic distances.

Most of the detected bacteria are known constituents of seawater or marine sediments (Buchan et al., 2005; Zhao et al., 2009; Bowman, 2014; Garcia and Müller, 2014; Gomez-Gil et al., 2014; Lastovica et al., 2014; López-Pérez and Rodriguez-Valera, 2014) and were likely associated with the sampled pharynges due to the ascidians' filter feeding. However, members of the *Vibrionaceae* (Gomez-Gil et al., 2014) and the genus *Endozoicomonas* (Kurahashi and Yokota, 2007; Yang et al., 2010; Nishijima et al., 2013; Pike et al., 2013; Hyun et al., 2014; Appolinario et al., 2016) have also been detected in association with other marine animals and thereby may represent bacteria with a more stable association with the sampled ascidians. The genus *Endozoicomonas* is of special interest as bacteria from this clade are only very rarely detected outside marine animals; only five sequences out of more than 1000 publically available *Endozoicomonas* 16S rRNA gene sequences originate from a non-marine-animal source (this study; Figure 1). This result and the detection of *Endozoicomonas* in 12 of the 17 initially screened ascidian specimens (culturing approach: 2/5, culture-independent approach: 10/12) indicated a symbiotic interaction between *Endozoicomonas* and ascidians, and consequently led us to focus this study on exploring this interaction.

### Distribution and prevalence of *Endozoicomonas* in ascidians

*Endozoicomonas* have previously been detected in specimens of the ascidians *Botrylloides leachi, Botrylloides* sp., *B. schlosseri, C. intestinalis, C. robusta, Ciona savignyi, Cystodytes dellechiajei, Diazona violacea, Didemnum* sp., *Eudistoma* sp., *Phallusia philippinensis, Polyclinella azemai*, and *Pycnoclavella diminuta* (Table 3). Using a newly designed specific PCR assay, we confirmed the presence of *Endozoicomonas* in *C. intestinalis* and *B. schlosseri*, and could expand their known host range to include: *A. aspersa, A. scabra, S. clava* and two Scandinavian *Ascidia* and *Ascidiella* species (Table 3). In the present study, a total of 61 ascidian specimens were screened; 54 of these yielded isolates or sufficient PCR product to confirm the presence of *Endozoicomonas* (Table S4). Among all screened ascidian species to date, *Endozoicomonas* has been detected in 25 to 100% of all specimens within a species; *Endozoicomonas* could be detected in 19 out of 54 host species (Table 3; for full details see Table S3). Overall, our data and analyses suggest that *Endozoicomonas* is a facultative symbiont of ascidians. Ascidians associated with *Endozoicomonas* have been detected in the Atlantic, Pacific, the Mediterranean, and Scandinavian waters (Table 3). This supports an earlier study, which suggested a cosmopolitan distribution of *Endozoicomonas* (Dishaw et al., 2014).

**Table 3:** **Distribution and prevalence of ascidian-associated ***Endozoicomonas*****.

**Ascidian host species^a^**	**PCR-based survey**	**Culturing**	**Specific PCR**	**DGGE**	**Overall**	**Overall [%]**	**Sampling location**	**References**
*Ascidia* sp.	3/6	0/2	7/12	–	10/20	50%	Gullmarsfjorden, Sweden	This study
*Ascidiella aspersa*	2/3	0/1	–	–	2/4	50%	Western Mediterranean, Spain; Gullmarsfjorden, Sweden	This study
*Ascidiella scabra*	4/4	1/1	7/7	–	12/12	100%	Gullmarsfjorden, Sweden	This study
*Ascidiella* sp.	–	1/1	4/4	–	4/4^b^	100%	Gullmarsfjorden, Sweden	This study
*Botrylloides leachi*^c^	Present	–	–	–	n.a.	n.a.	New Zealand	Cahill et al., 2016
*Botrylloides* sp.	1/1	–	–	–	1/1	100%	Southern California, USA	Tianero et al., 2014
*Botryllus schlosseri*	1/1	–	6/6	–	6/6^d^	100%	Limfjorden, Denmark; Gullmarsfjorden, Sweden	This study
*Botryllus schlosseri*^c^	Present	–	–	–	n.a.	n.a.	New Zealand	Cahill et al., 2016
*Ciona intestinalis*	7/7	–	–	–	7/7	100%	Cape Cod, USA; Southern California, USA; Fusaro Lake, Italy	Dishaw et al., 2014
*Ciona intestinalis*	0/1	–	3/3	–	3/4	75%	Gullmarsfjorden, Sweden	This study
*Ciona robusta*^c^	Present	–	–	–	n.a.	n.a.	New Zealand	Cahill et al., 2016
*Ciona savignyi*^c^	Present	–	–	–	n.a.	n.a.	New Zealand	Cahill et al., 2016
*Cystodytes dellechiajei*^c^	Present	–	–	–	n.a.	n.a.	Western Mediterranean, Spain	Martínez-García et al., 2007
*Diazona violacea*	–	–	–	1/3	1/3	33%	Western Mediterranean, Spain	Martínez-García et al., 2010
*Didemnum* sp.	–	1/1	–	–	1/1	100%	North coast of São Paulo state, Brazil	Menezes et al., 2010
*Didemnum* sp.	4/10	–	–	–	4/10	40%	Southern California, USA; Papua New Guinea	Tianero et al., 2014
*Eudistoma* sp.	2/2	–	–	–	2/2	100%	Florida Keys, USA	Tianero et al., 2014
*Phallusia philippinensis*	1/1	–	–	–	1/1	100%	Great Barrier Reef, Australia	Erwin et al., 2014
*Polyclinella azemai*	–	–	–	1/2	1/2	50%	Western Mediterranean, Spain	Martínez-García et al., 2010
*Pycnoclavella diminuta*	1/3	–	–	–	1/3	33%	Great Barrier Reef, Australia	Erwin et al., 2014
*Styela clava*	–	–	1/4	–	1/4	25%	Limfjorden, Denmark	This study

*The number of Endozoicomonas–positive specimens relative to all screened specimens is shown for each ascidian species. −, no data; ND, not determined*.

a *Ascidian species in which Endozoicomonas has not been detected so far (for details see Table S3): Aplousobranchia: Aplidium protectans, Aplidium sp., Clavelina arafurensis, Clavelina meridionalis, Cystodytes sp., Didemnum cf. albopunctatum, Didemnum cf. granulatum, Didemnum fulgens, Didemnum multispirale, Didemnum sp., Eudistoma amplum, Leptoclinides madara, Lissoclinum badium, Lissoclinum bistratum, Lissoclinum cf. caspulatum, Lissoclinum patella, Polycitor giganteus, Pseudodistoma crucigaster, Pycnoclavella sp., Synoicum castellatum, Trididemnum sp.; Phlebobranchia: Ascidia sp., Ecteinascidia diaphanis, Ecteinascidia turbinata, Perophora aff. modificata, Phallusia arabica, Phallusia julinea; Stolidobranchia: Botrylloides violaceus, Molgula manhattensis, Polycarpa argentata, Polycarpa aurata, Pyura sp., Styela plicata, Styela sp*.

b *One specimen was screened both by culturing and specific PCR*.

c *Based on the publication, no prevalence data could be inferred*.

d *One specimen was screened both during the PCR-based survey and by specific PCR*.

### An ascidian-specific *Endozoicomonas* clade

Phylogenetic analysis of the *Endozoicomonas* clade was complicated by the observation that its members can harbor multiple divergent paralogs of the 16S rRNA gene (Figure S2). Even after focusing on the dominant paralog present in public databases (paralog-1 type), the phylogeny of the clade still remains largely unresolved. Phylogenies reconstructed using ML, MP, and BI-approaches were highly divergent as evidenced by relative Robinson-Foulds values (which represent the percentage of splits that are unique to one of the two compared trees) between 0.65 and 0.71 and a highly ambiguous (i.e., multifurcating) consensus phylogeny (Figure 1).

**Figure 2:**
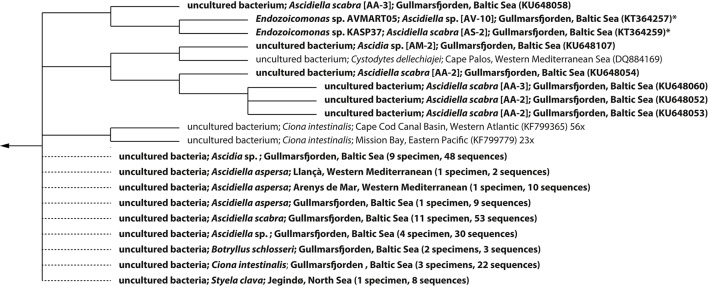
**Dendrogram of 16S rRNA gene sequences of ***Endozoicomonas*** affiliating with the ascidian-specific subclade depicted in Figure 1**. Host species and geographic origin are given for each sequence, followed by the corresponding accession number in brackets. Sequences generated in the present study are shown in bold face. Sequences originating from *Endozoicomonas* isolates are marked with an asterisk. The dendrogram was truncated and does not show an additional 77 sequences of bacteria associated with Atlantic and Pacific specimens of the ascidian *Ciona intestinalis* (all from Dishaw et al., 2014). A summary of partial *Endozoicomonas* sequences generated in this study and confidently assigned to the ascidian-specific subclade was added manually (indicated with dotted lines) to illustrate host and geographic distribution. For these, the number of specimens harboring representatives of the ascidian-specific subclade as well as the total number of positively assigned sequences is shown in brackets (see also Table S6). Branch lengths do not represent phylogenetic distances.

Despite divergent tree topologies, several host-specific subclades were consistently detected (Figure 1; Figures S4–S6). Most relevant to this study and with the exception of one of the two sequences originating from the Mediterranean *C. dellechiajei*, all ascidian-derived *Endozoicomonas* nearly full-length sequences formed a newly defined, ascidian-specific subclade (Figures 1, 2). This subclade contained 88 ascidian-derived sequences: eight originating from Baltic Sea *Ascidia* and *Ascidiella* species (this study), one originating from a Mediterranean specimen of *C. dellechiajei* (Martínez-García et al., 2007) and 79 originating from Atlantic and Pacific specimens of *C. intestinalis* (Dishaw et al., 2014; Figure 2). Pairwise sequence identities within the subclade were as low as 96.8% (Table S5); this indicates a clade at the genus level (Yarza et al., 2014) containing different species. The most closely related species to this subclade are *Endozoicomonas atrinae* (isolated from the intestine of the marine pen shell *Atrina pectinata*) and *Endozoicomonas elysicola* (isolated from the marine sea slug *Elysia ornata*) with sequence identities to the ascidian-specific subclade of 96.6–98.1 and 96.5–98.0%, respectively (Table S5). The majority of generated partial *Endozoicomonas* 16S rRNA gene sequences (61%; 185 out of 301 sequences) could be confidently assigned to the ascidian-specific subclade (Figure 2; Table S6). However, for sequences obtained from *B. schlosseri*, the only colonial ascidian tested in the present study, overall only 11% (3 out of 28) of the retrieved *Endozoicomonas* sequences affiliated with the ascidian-specific subclade (Table S6). Other noteworthy cases include two specimens of *Ascidia* sp. (specimens AM-5 and AV-12) and two specimens of *A. scabra* (AA-12 and AS-3), where also the majority of *Endozoicomonas* sequences (>70%) did not affiliate with the ascidian-specific subclade (Table S6). This result may suggest a larger diversity of ascidian-associated *Endozoicomonas* (possibly divided into ascidian specialists and more generalist species), or simply be due to insufficient phylogenetic information.

The current data set is insufficient to explore host-species specificity within the ascidian-specific subclade due to the limited number of nearly full-length 16S rRNA gene sequences of ascidian-derived *Endozoicomonas*, the limited resolution of the 16S rRNA gene, and the presence of divergent paralogs, which complicates the analysis of environmental sequences. The question of a species-specific symbiosis thus has to await full genome information for multiple ascidian-derived *Endozoicomonas* strains.

### Interaction between *Endozoicomonas* and ascidians

Whether *Endozoicomonas* are transmitted horizontally or vertically between ascidians is currently not clear. A previous study targeting bacteria associated with the colonial ascidian *C. dellechiajei* detected the presence of *Endozoicomonas* in adult specimens but not in larvae (Martínez-García et al., 2007). Unfortunately, no ascidian larvae or juveniles could be obtained for the present study. However, the detection of a sequence affiliating with the ascidian-specific subclade in a water sample from the Gullmarsfjord sampling site (accession number: KU648384; this study) suggests dissemination of *Endozoicomonas* by horizontal transmission.

**Figure 3:**
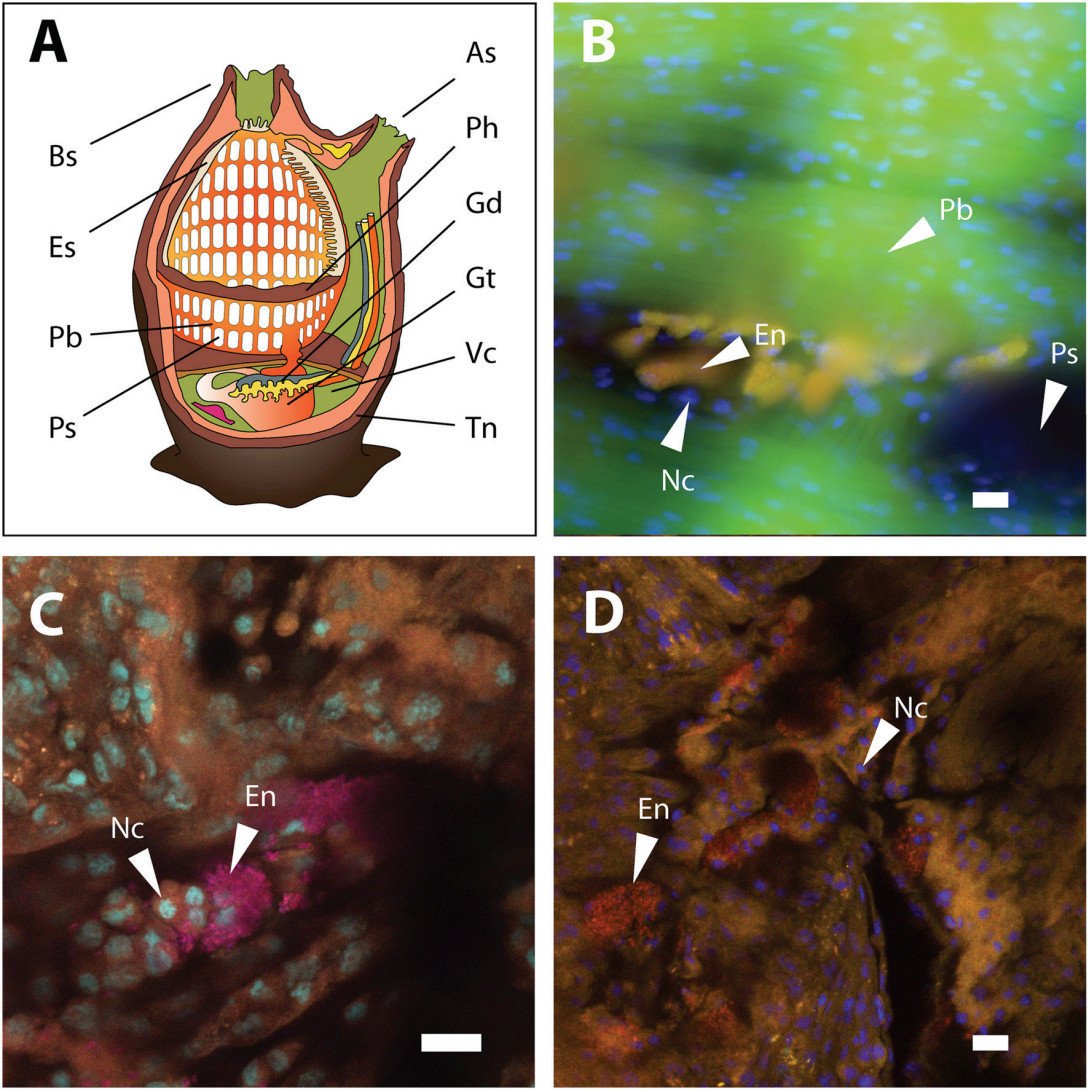
**Ascidian anatomy and FISH detection of ***Endozoicomonas*** in the pharynx tissue of ***A. aspersa***. (A)** Anatomical sketch of a solitary ascidian (redrawn from multiple sources). **(B)** Micrograph of *Endozoicomonas* microcolonies detected by probe ENDO-1240 (green) and probe mix EUB338 I-III (red). Overlay of the two probes produced the yellow-colored colonies observed in the micrograph. Autofluorescent pharynx tissue is shown in green. **(C)** Confocal micrograph of pharynx-associated *Endozoicomonas* microcolonies detected by probe ENDO-580 (red) and probe mix EUB338 I-III (pink). Overlay of the two probes produced the magenta-colored colonies shown in the micrograph. Pharynx nuclei were stained with DAPI (cyan). **(D)** Confocal micrograph of pharynx-associated *Endozoicomonas* microcolonies detected by probe ENDO-580 (red). Pharynx nuclei were stained with DAPI (blue). All scale bars, 10 5m. As, atrial siphon; Bs, buccal siphon; En, Endozoicomonas microcolonies; Es, endostyle; Gd, gonads; Gt, gut; Nc, nuclei of pharynx tissue; Pb, pharyngeal bars; Ph, pharynx; Ps, pharyngeal stigmata; Tn, tunic; Vc, visceral cavity.

*Endozoicomonas* cells were detected in pharynx samples of *A. aspersa* (Figure 3), *A. scabra*, and *Ascidia* sp. (one specimen each) by FISH. *Endozoicomonas* formed microcolonies on the pharyngal epithelium outside of the host cells. However, due to high background fluorescence of the pharynx tissue, an additional intracellular localization within the host's pharynx cells cannot be excluded. Host cell nuclei and *Endozoicomonas* microcolonies did never co-localize (Figures 3C,D), rendering an intranuclear localization of *Endozoicomonas* (as in bathymodiolin mussels) unlikely. These FISH results suggest that *Endozoicomonas* are not just enriched by the ascidians from seawater by filtration but are actually able to grow *in situ* in their host, where they occupy a protected niche in crevices and grooves of the pharynx. Currently no data exist on the localization of *Endozoicomonas* in other ascidian species. However, similar bacterial microcolonies, albeit of unknown identity, have previously been reported in association with the pharynx of larvae of the ascidian *Ecteinascidia turbinata* (Moss et al., 2003). *Endozoicomonas* associated with the Red Sea coral *Stylophora pistillata* also grow as microcolonies in the coral endoderm (Bayer et al., 2013), while *Endozoicomonas* associated with bathymodiolin mussels grow inside cell nuclei of the host (Zielinski et al., 2009). Interestingly, in both ascidians and the coral, *Endozoicomonas* assumes a smaller cell size (diameter, 1 μm) and coccoid morphology *in situ*, compared to the larger rod-shaped cells (cell size up to 0.5 × 10 μm) of laboratory pure cultures (Kurahashi and Yokota, 2007; Yang et al., 2010; Nishijima et al., 2013; Pike et al., 2013; Hyun et al., 2014; Figure S7, this study), indicating morphological adaptation to the host environment.

Putative interactions between *Endozoicomonas* and ascidians were investigated based on two *Endozoicomonas* isolates affiliating with the ascidian-specific subclade; *Endozoicomonas* sp. AVMART05 and KASP37 (Table 4; Table S2). Theses isolates were obtained from *Ascidiella scabra* and *Ascidiella* sp., respectively, on marine agar (containing yeast extract and peptone), and were thus characterized as chemoorganoheterotrophs. Since the intranuclear *Endozoicomonas* of bathymodiolin mussels were proposed to use the host's chromatin as nutritional source (Zielinski et al., 2009), the possibility of a similar lifestyle was tested for the ascidian-associated *Endozoicomonas*. In support of the chromatin-feeding hypothesis, both isolates showed the production of extracellular DNase (Table 4). However, the isolates were not able to metabolize high-molecular weight DNA or dNTP's (Table 4). These results and the apparent extracellular localization of ascidian-associated *Endozoicomona*s indicate a different kind of interaction with the host compared to the intranuclear mussel parasites.

**Table 4:** **Physiological characteristics of ***Endozoicomonas*** isolated from ascidians and ***E. elysicola*****.

**Physiological property**	***Endozoicomonas* sp. AVMART05**	***Endozoicomonas* sp. KASP37**	***E. elysicola***
Hemolysis	–	–	n.d.
Antibacterial activity: diffusion assay against *E.coli*	–	–	n.d.
Antibacterial activity: diffusion assay against *B. cereus*	–	–	n.d.
Antibacterial activity: diffusion assay against *S. epidermidis*	–	–	n.d.
Antibacterial activity: overlay assay against *E.coli*	–	–	n.d.
Antibacterial activity: overlay assay against *B. cereus*	–	–	n.d.
Antibacterial activity: overlay assay against *S. epidermidis*	–	–	n.d.
DNase activity	+	+	+
Metabolizing of salmon sperm DNA	–	–	n.d.
Metabolizing of dNTP's	–	–	n.d.
Growth on mucin	+	+	+

*All data was obtained in the present study. Abbreviations: −, negative; +, positive; n.d., no data*.

The ascidian-specific *Endozoicomonas* subclade contains a sequence retrieved from *C. dellechiajei*, an ascidian shown to produce bioactive compounds (Loukaci et al., 2000; López-Legentil et al., 2005; Bontemps et al., 2010). Based on this and the observation of antibacterial properties of *Endozoicomonas* isolated from marine sponges (Gram et al., 2010; Flemer et al., 2012; Rua et al., 2014), we hypothesized that *Endozoicomonas* defend the host against bacterial infections or predators in a mutualistic relationship. In our assays, neither of the two ascidian-derived isolates showed evidence of antibacterial activity, or the production of cytotoxic compounds that could indicate a predator-deterrent role (Lopanik, 2014). As the production of cytotoxins is also a common trait of pathogens (Aktories and Barbieri, 2005), the lack of cytotoxic activity does not support a pathogenic interaction between *Endozoicomonas* and ascidians either. However, this does not exclude the production of secondary metabolites with functions not assessed here or produced only under *in situ* conditions, i.e., when associated with the ascidian host.

The majority of described ascidian species are filter feeders that catch their food in a moving mucus layer covering their pharynx that is continuously secreted by the endostyle (MacGinitie, 1939; Bone et al., 2003). The ascidian mucus layer apparently consists of mucopolysaccharides surrounding a protein core and it is currently not known if it is similar across different ascidians (Flood and Fiala-Medioni, 1981; Bone et al., 2003). Both ascidian-derived isolates grew well on porcine mucus glycoproteins (mucin) as substrate (Table 4), indicating that mucus may be an important nutrient source for *Endozoicomonas* in ascidians. Interestingly, *E. elysicola* also grew well on mucin, suggesting that this trait may be more widespread among the *Endozoicomonas* clade.

## Conclusion

Based on our combined molecular screening, phylogenetic, FISH, and functional results, ascidian-associated *Endozoicomonas* appear to form a specific, yet facultative symbiosis with their host. They are likely horizontally transmitted commensals that live off the mucus continuously secreted by the pharynx without affecting the ascidian host. Additionally, based on the observations that the sea-slug-derived *E. elysicola* also grows on mucin (this study), and that *Endozoicomonas* symbionts are often found in mucus layers of other hosts (Morrow et al., 2012; Bayer et al., 2013; Carlos et al., 2013; Correa et al., 2013; Vezzulli et al., 2013), we propose that mucus-degradation and metabolism also plays a role in other *Endozoicomonas*-hosts systems.

## Author contributions

LS and AS jointly designed experiments. LS and AS wrote the manuscript. MO, AS, KK, JJ, and PF collected Swedish ascidians. SL collected Spanish ascidians. LS, JJ, PF, KK, and AS collected Danish ascidians. PF and MO assisted with ascidian identification and dissection. LS, JJ, and SL generated CO1 gene sequence data. SL performed phylogenetic analysis of ascidian CO1 genes. LS, JJ, KK, and AS generated sequence data of ascidian-associated bacteria. LS and KK isolated ascidian-associated bacteria. LS performed phylogenetic analyses, re-analyses of next generation sequencing data, and physiological tests of *Endozoicomonas* isolates. LS and JJ designed and tested *Endozoicomonas*-specific oligonucleotide primers and probes. AS performed fluorescence *in situ* hybridizations on pharynx sections. All co-authors commented on the manuscript.

## Funding

This project was funded by the Danish Council for Independent Research/Natural Sciences (DFF FNU; 09-064111), the Aarhus University Research Foundation (AU Ideas Program 2013; R9-A995-13-S833), the European Community (ASSEMBLE grant agreement no. 227799), the Danish National Research Foundation, and the Max Planck Society. Sampling in Spain was supported by the Spanish Ministry of Economy and Competitiveness (MINECO) project MARSYMBIOMICS CTM2013-43287-P.

### Conflict of interest statement

The authors declare that the research was conducted in the absence of any commercial or financial relationships that could be construed as a potential conflict of interest.
