# Supplementary material for: Endozoicomonas Are Specific, Facultative Symbionts of Sea Squirts
Source: Front Microbiol. 2016 Jul 12;7:1042. doi: 10.3389/fmicb.2016.01042 (PMC4940369; doi:10.3389/fmicb.2016.01042)
Supplement: Supplementary file 1 [file Table1.PDF]

Table S1. Metadata of screened ascidian specimens

Identity	Specimen identifier	Sampling date	Sampling site	Geo data (lat, lon)	Water depth	Comments
Ascidia species						
Ascidia sp.	AM-1	03-Sep-09	Gullmarsfjorden, Sweden	58.2658 N, 11.4175 E	n.d.	morphological identity: *A. mentula*
Ascidia sp.	AM-2	03-Sep-09	Gullmarsfjorden, Sweden	58.2658 N, 11.4175 E	n.d.	morphological identity: *A. mentula*
Ascidia sp.	AM-3	11-Jun-10	Gullmarsfjorden, Sweden; Station 1 - Stangholmgrundet	58.26567 N, 11.41750 E	22-30m	morphological identity: *A. mentula*
Ascidia sp.	AM-4	11-Jun-10	Gullmarsfjorden, Sweden; Station 1 - Stangholmgrundet	58.26567 N, 11.41750 E	22-30m	morphological identity: *A. mentula*
Ascidia sp.	AM-5	29-Nov-10	Gullmarsfjorden, Sweden	58.2658 N, 11.4175 E	n.d.	morphological identity: *A. mentula*
Ascidia sp.	AA-1	09-Jun-09	Gullmarsfjorden, Sweden	58.2658 N, 11.4175 E	n.d.	
Ascidia sp.	AA-4	09-Jun-09	Gullmarsfjorden, Sweden	58.2658 N, 11.4175 E	n.d.	
Ascidia sp.	AA-5	11-Jun-10	Gullmarsfjorden, Sweden; Station 1 - Stangholmgrundet	58.26567 N, 11.41750 E	22-30m	
Ascidia sp.	AA-6	11-Jun-10	Gullmarsfjorden, Sweden; Station 1 - Stangholmgrundet	58.26567 N, 11.41750 E	22-30m	
Ascidia sp.	AA-8	11-Jun-10	Gullmarsfjorden, Sweden; Station 2 - Staangehuvud	58.27083 N, 11.41250 E	16m	
Ascidia sp.	AA-9	11-Jun-10	Gullmarsfjorden, Sweden; Station 2 - Staangehuvud	58.27083 N, 11.41250 E	16m	
Ascidia sp.	AV-1	03-Sep-09	Gullmarsfjorden, Sweden	58.2658 N, 11.4175 E	n.d.	
Ascidia sp.	AV-11	24-Sep-11	Gullmarsfjorden, Sweden	58.2658 N, 11.4175 E	n.d.	
Ascidia sp.	AV-12	24-Sep-11	Gullmarsfjorden, Sweden	58.2658 N, 11.4175 E	n.d.	
Ascidia sp.	AV-2	03-Sep-09	Gullmarsfjorden, Sweden	58.2658 N, 11.4175 E	n.d.	
Ascidia sp.	AV-3	11-Jun-10	Gullmarsfjorden, Sweden; Station 1 - Stangholmgrundet	58.26567 N, 11.41750 E	22-30m	
Ascidia sp.	AV-4	11-Jun-10	Gullmarsfjorden, Sweden; Station 1 - Stangholmgrundet	58.26567 N, 11.41750 E	22-30m	
Ascidia sp.	AV-5	11-Jun-10	Gullmarsfjorden, Sweden; Station 1 - Stangholmgrundet	58.26567 N, 11.41750 E	22-30m	
Ascidia sp.	AV-6	11-Jun-10	Gullmarsfjorden, Sweden; Station 2 - Staangehuvud	58.27083 N, 11.41250 E	16m	
Ascidia sp.	AV-7	11-Jun-10	Gullmarsfjorden, Sweden; Station 2 - Staangehuvud	58.27083 N, 11.41250 E	16m	
Ascidia sp.	AV-8	11-Jun-10	Gullmarsfjorden, Sweden; Station 2 - Staangehuvud	58.27083 N, 11.41250 E	16m	
Ascidia sp.	AV-9	11-Jun-10	Gullmarsfjorden, Sweden; Station 2 - Staangehuvud	58.27083 N, 11.41250 E	16m	
Ascidia sp.	AJ-2	24-Sep-11	Gullmarsfjorden, Sweden	58.2658 N, 11.4175 E	n.d.	Due to small specimen size whole animal was sampled

Table S1. continued from previous page

Identity	Specimen identifier	Sampling date	Sampling site	Geo data (lat, lon)	Water depth	Comments
Ascidiella species						
Ascidiella sp.	AJ-1	24-Sep-11	Gullmarsfjorden, Sweden	58.2658 N, 11.4175 E	n.d.	
Ascidiella sp.	AJ-3	24-Sep-11	Gullmarsfjorden, Sweden	58.2658 N, 11.4175 E	n.d.	
Ascidiella sp.	AJ-4	24-Sep-11	Gullmarsfjorden, Sweden	58.2658 N, 11.4175 E	n.d.	
Ascidiella sp.	AV-10	29-Nov-10	Gullmarsfjorden, Sweden	58.2658 N, 11.4175 E	n.d.	
Ascidiella aspersa						
A. aspersa	AA-14	01-Feb-13	Llançà, Catalunya, Spain	42.36667 N, 3.15000 E	n.d.	previously published by Lopez-Legentil et al., 2015a as specimen LLAN-SC
A. aspersa	AA-15	19-Jan-13	Arenys de Mar, Catalunya, Spain	41.57167 N, 2.55500 E	n.d.	previously published by Lopez-Legentil et al., 2015a as specimen AM-2; sampled gut together with pharynx due to small specimen size
A. aspersa	AA-16	31-Jan-13	Empuriabrava, Catalunya, Spain	42.24333 N, 3.13500 E	n.d.	previously published by Lopez-Legentil et al., 2015a as specimen EMB-AS
A. aspersa	AS-1	29-Nov-10	Gullmarsfjorden, Sweden	58.2658 N, 11.4175 E	n.d.	
Ascidiella scabra						
A. scabra	AA-11	11-Jun-10	Gullmarsfjorden, Sweden; Station 2 - Staangehuvud	58.27083 N, 11.41250 E	16m	
A. scabra	AA-7	11-Jun-10	Gullmarsfjorden, Sweden; Station 1 - Stangholmgrundet	58.26567 N, 11.41750 E	22-30m	
A. scabra	AA-10	11-Jun-10	Gullmarsfjorden, Sweden; Station 2 - Staangehuvud	58.27083 N, 11.41250 E	16m	
A. scabra	AA-12	24-Sep-11	Gullmarsfjorden, Sweden	58.2658 N, 11.4175 E	n.d.	
A. scabra	AA-13	24-Sep-11	Gullmarsfjorden, Sweden	58.2658 N, 11.4175 E	n.d.	
A. scabra	AA-2	09-Jun-09	Gullmarsfjorden, Sweden	58.2658 N, 11.4175 E	n.d.	
A. scabra	AA-3	09-Jun-09	Gullmarsfjorden, Sweden	58.2658 N, 11.4175 E	n.d.	
A. scabra	AS-2	24-Sep-11	Gullmarsfjorden, Sweden	58.2658 N, 11.4175 E	n.d.	
A. scabra	AS-3	24-Sep-11	Gullmarsfjorden, Sweden	58.2658 N, 11.4175 E	n.d.	
A. scabra	AS-4	24-Sep-11	Gullmarsfjorden, Sweden	58.2658 N, 11.4175 E	n.d.	
A. scabra	AS-5	24-Sep-11	Gullmarsfjorden, Sweden	58.2658 N, 11.4175 E	n.d.	

Table S1. continued from previous page

Identity	Specimen identifier	Sampling date	Sampling site	Geo data (lat, lon)	Water depth	Comments
Botryllus schlosseri						
B. schlosseri	BS-1	29-Jun-11	Jegindø Harbour, Denmark	56.65200 N, 8.63717 E	1m	Whole animal was sampled
B. schlosseri	BS-2	24-Sep-11	Gullmarsfjorden, Sweden	58.2658 N, 11.4175 E	n.d.	Whole animal was sampled
B. schlosseri	BS-3	24-Sep-11	Gullmarsfjorden, Sweden	58.2658 N, 11.4175 E	n.d.	Whole animal was sampled
B. schlosseri	BS-4	24-Sep-11	Gullmarsfjorden, Sweden	58.2658 N, 11.4175 E	n.d.	Whole animal was sampled
B. schlosseri	BS-5	24-Sep-11	Gullmarsfjorden, Sweden	58.2658 N, 11.4175 E	n.d.	Whole animal was sampled
B. schlosseri	BS-6	24-Sep-11	Gullmarsfjorden, Sweden	58.2658 N, 11.4175 E	n.d.	Whole animal was sampled
Ciona intestinalis						
C. intestinalis	CI-1	09-Jun-09	Gullmarsfjorden, Sweden	58.2658 N, 11.4175 E	n.d.	
C. intestinalis	CI-2	09-Jun-09	Gullmarsfjorden, Sweden	58.2658 N, 11.4175 E	n.d.	
C. intestinalis	CI-3	03-Sep-09	Gullmarsfjorden, Sweden	58.2658 N, 11.4175 E	n.d.	
C. intestinalis	CI-4	26-Sep-09	Limfjorden	56.70550 N, 9.18717 E	n.d.	
C. intestinalis	CI-5	11-Jun-10	Gullmarsfjorden, Sweden; Station 1 - Stangholmgrundet	58.26567 N, 11.41750 E	22-30m	
C. intestinalis	CI-6	11-Jun-10	Gullmarsfjorden, Sweden; Station 1 - Stangholmgrundet	58.26567 N, 11.41750 E	22-30m	
C. intestinalis	CI-7	24-Sep-11	Gullmarsfjorden, Sweden	58.2658 N, 11.4175 E	n.d.	
C. intestinalis	CI-8	24-Sep-11	Gullmarsfjorden, Sweden	58.2658 N, 11.4175 E	n.d.	
C. intestinalis	CI-9	24-Sep-11	Gullmarsfjorden, Sweden	58.2658 N, 11.4175 E	n.d.	
Styela clava						
S. clava	SC-1	29-Jun-11	Jegindø Harbour, Denmark	56.65200 N, 8.63717 E	1m	
S. clava	SC-2	29-Jun-11	Jegindø Harbour, Denmark	56.65200 N, 8.63717 E	1m	
S. clava	SC-3	29-Jun-11	Jegindø Harbour, Denmark	56.65200 N, 8.63717 E	1m	
S. clava	SC-4	29-Jun-11	Jegindø Harbour, Denmark	56.65200 N, 8.63717 E	1m	
Water samples						
Water sample	D_water	24-Sep-11	Gullmarsfjorden, Sweden	58.2658 N, 11.4175 E	n.d.	used c. 0.5 L for DNA extraction
Water sample	W_18m	24-Sep-11	Gullmarsfjorden, Sweden; Station Hisäcker	56.26967 N, 11.41317 E	18m	used c. 0.5 L for DNA extraction
Water sample	W_30m	24-Sep-11	Gullmarsfjorden, Sweden; Station 1 - Stangholmgrundet	58.26567 N, 11.41750 E	22-30m	used c. 0.5 L for DNA extraction
Water sample	W_Oscar	24-Sep-11	Gullmarsfjorden, Sweden	58.2658 N, 11.4175 E	n.d.	used c. 0.5 L for DNA extraction
