# Supplementary material for: Endozoicomonas Are Specific, Facultative Symbionts of Sea Squirts
Source: Front Microbiol. 2016 Jul 12;7:1042. doi: 10.3389/fmicb.2016.01042 (PMC4940369; doi:10.3389/fmicb.2016.01042)
Supplement: Supplementary file 2 [file Table2.PDF]

Table S2. Metadata of ascidian-derived isolates

Isolate, identifier	Isolate, accession number	Source, host	Source, isolation temperature	Source, isolation medium	Closest relative, name	Closest relative, accession number	Closest relative, nucleotide identity [%]
KASP35	n.d.	*A. scabra* AS-2	RT	50% MA	Could not be identifed	n.d.	n.d.
KASP36	n.d.	*A. scabra* AS-2	RT	50% MA	Could not be identifed	n.d.	n.d.
KASP37	KT364258, KT364259, KT364260	*A. scabra* AS-2	RT	50% MA	*Endozoicomonas elysicola* strain MKT110	NR_041264	96.62-97.08*
KASP38	n.d.	*A. scabra* AS-2	RT	50% MA	Could not be identifed	n.d.	n.d.
KASP39	n.d.	*A. scabra* AS-2	RT	50% MA	Could not be identifed	n.d.	n.d.
KASP40	n.d.	*A. scabra* AS-2	RT	50% MA	Could not be identifed	n.d.	n.d.
KASP41	n.d.	*A. scabra* AS-2	RT	50% MA	Could not be identifed	n.d.	n.d.
KASP42	n.d.	*A. scabra* AS-2	RT	50% MA	Could not be identifed	n.d.	n.d.
KASP43	n.d.	*A. scabra* AS-2	RT	50% MA	Could not be identifed	n.d.	n.d.
KASP44	n.d.	*A. scabra* AS-2	RT	50% MA	Could not be identifed	n.d.	n.d.
KASP45	n.d.	*A. scabra* AS-2	RT	50% MA	Could not be identifed	n.d.	n.d.
KASP46	n.d.	*A. scabra* AS-2	RT	50% MA	Could not be identifed	n.d.	n.d.
KASP47	n.d.	*A. scabra* AS-2	RT	50% MA	Could not be identifed	n.d.	n.d.
KASP48	n.d.	*A. scabra* AS-2	RT	50% MA	Could not be identifed	n.d.	n.d.
KASP1	KU647904	*Ascidia* sp. AV-11	14°C	50% MA	*Colwellia aestuarii* strain SMK-10	NR_043509	98.77
KASP2	n.d.	*Ascidia* sp. AV-11	14°C	50% MA	Could not be identifed	n.d.	n.d.
KASP3	KU647905	*Ascidia* sp. AV-11	14°C	50% MA	*Pseudoalteromonas carrageenovora* strain NBRC 12985	NR_113605	99.57
KASP4	KU647906	*Ascidia* sp. AV-11	14°C	50% MA	*Shewanella piezotolerans* strain WP3	NR_074738	98.65
KASP5	KU647907	*Ascidia* sp. AV-11	14°C	50% MA	*Vibrio gallaecicu*s strain CECT 7244	NR_044520	99.37
KASP6	KU647908	*Ascidia* sp. AV-11	14°C	50% MA	*Shewanella piezotolerans* strain WP3	NR_074738	98.65
KASP7	KU647909	*Ascidia* sp. AV-11	14°C	50% MA	*Shewanella piezotolerans* strain WP3	NR_074738	98.65
KASP8	KU647910	*Ascidia* sp. AV-11	14°C	50% MA	*Shewanella colwelliana* strain ATCC 39565	NR_043074	99.79
KASP9	KU647911	*Ascidia* sp. AV-11	14°C	50% MA	*Shewanella piezotolerans* strain WP3	NR_074738	98.65
KASP10	KU647912	*Ascidia* sp. AV-11	14°C	50% MA	*Shewanella marinintestina* strain IK-1	NR_024791	100
KASP11	KU647913	*Ascidia* sp. AV-11	14°C	50% MA	*Shewanella japonica* strain NBRC 103171	NR_114234	98.63
KASP12	KU647914	*Ascidia* sp. AV-11	14°C	50% MA	*Shewanella colwelliana* strain ATCC 39565	NR_043074	99.86
KASP13	KU647915	*Ascidia* sp. AV-11	14°C	50% MA	*Shewanella piezotolerans* strain WP3	NR_074738	98.58
KASP14	n.d.	*Ascidia* sp. AV-11	RT	50% MA	Could not be identifed	n.d.	n.d.
KASP15	n.d.	*Ascidia* sp. AV-11	RT	50% MA	Could not be identifed	n.d.	n.d.
KASP16	n.d.	*Ascidia* sp. AV-11	RT	50% MA	Could not be identifed	n.d.	n.d.

Table S2. continued from previous page

Isolate, identifier	Isolate, accession number	Source, host	Source, isolation temperature	Source, isolation medium	Closest relative, name	Closest relative, accession number	Closest relative, nucleotide identity [%]
KASP17	n.d.	*Ascidia* sp. AV-11	RT	50% MA	Could not be identifed	n.d.	n.d.
KASP18	n.d.	*Ascidia* sp. AV-11	RT	50% MA	Could not be identifed	n.d.	n.d.
KASP19	n.d.	*Ascidia* sp. AV-11	RT	50% MA	Could not be identifed	n.d.	n.d.
KASP20	KU647916	*Ascidia* sp. AV-11	RT	50% MA	*Shewanella piezotolerans* strain WP3	NR_074738	98.65
KASP21	KU647917	*Ascidia* sp. AV-11	RT	50% MA	*Shewanella piezotolerans* strain WP3	NR_074738	98.65
KASP22	KU647918	*Ascidia* sp. AV-11	RT	50% MA	*Vibrio crassostreae* strain LGP 7	NR_044078	97.78
KASP23	KU647919	*Ascidia* sp. AV-11	RT	50% MA	*Shewanella piezotolerans* strain WP3	NR_074738	98.65
KASP24	KU647920	*Ascidia* sp. AV-11	RT	50% MA	*Shewanella colwelliana* strain ATCC 39565	NR_043074	99.86
KASP25	KU647921	*Ascidia* sp. AV-11	RT	50% MA	*Vibrio hemicentroti* strain AlyHP32	NR_109668	99.3
KASP26	KU647922	*Ascidia* sp. AV-11	RT	50% MA	*Vibrio gigantis* strain LGP 13	NR_044079	99.23
KASP27	KU647923	*Ascidia* sp. AV-11	RT	50% MA	*Shewanella piezotolerans* strain WP3	NR_074738	98.65
KASP28	KU647924	*Ascidia* sp. AV-11	RT	50% MA	*Vibrio gigantis* strain LGP 13	NR_044079	98.59
KASP29	KU647925	*Ascidia* sp. AV-11	RT	50% MA	*Shewanella piezotolerans* strain WP3	NR_074738	98.65
KASP30	KU647926	*Ascidia* sp. AV-11	RT	50% MA	*Pseudoalteromonas carrageenovora* strain NBRC 12985	NR_113605	99.57
KASP31	KU647927	*Ascidia* sp. AV-11	RT	50% MA	*Vibrio crassostreae* strain LGP 7	NR_044078	99.58
KASP32	KU647928	*Ascidia* sp. AV-11	RT	50% MA	*Shewanella piezotolerans* strain WP3	NR_074738	98.65
KASP33	KU647929	*Ascidia* sp. AV-11	RT	50% MA	*Vibrio gallaecicus* strain CECT 7244	NR_044520	99.51
KASP34	KU647930	*Ascidia* sp. AV-11	RT	50% MA	*Pseudoalteromonas ulvae* strain UL12	NR_025032	99.93
AA500401	KU647850	*Ascidia* sp. AM-5	4°C	50% MA	*Shewanella fidelis* strain KMM 3582	NR_025195	99.46
AA500402	KU647851	*Ascidia* sp. AM-5	4°C	50% MA	*Vibrio splendidus* LGP32 strain LGP32	NR_074953	99.88
AA500403	KU647852	*Ascidia* sp. AM-5	4°C	50% MA	*Shewanella fidelis* strain KMM 3582	NR_025195	99.45
AA500404	KU647853	*Ascidia* sp. AM-5	4°C	50% MA	*Shewanella fidelis* strain KMM 3582	NR_025195	99.44
AA501001	KU647854	*Ascidia* sp. AM-5	10°C	50% MA	*Vibrio splendidus* LGP32 strain LGP32	NR_074953	99.88
AA501002	KU647855	*Ascidia* sp. AM-5	10°C	50% MA	*Vibrio splendidus* LGP32 strain LGP32	NR_074953	99.54
AA501003	KU647856	*Ascidia* sp. AM-5	10°C	50% MA	*Photobacterium frigidiphilum* strain SL13	NR_042964	99.89
AA501004	KU647857	*Ascidia* sp. AM-5	10°C	50% MA	*Shewanella fidelis* strain KMM 3582	NR_025195	96.71
AA501005	KU647858	*Ascidia* sp. AM-5	10°C	50% MA	*Moritella marina* strain ATCC 15381	NR_040842	98.72
AA501006	KU647859	*Ascidia* sp. AM-5	10°C	50% MA	*Shewanella fidelis* strain KMM 3582	NR_025195	99.46
AA501501	KU647860	*Ascidia* sp. AM-5	15°C	50% MA	*Vibrio splendidus* LGP32 strain LGP32	NR_074953	99.42
AA501502	KU647861	*Ascidia* sp. AM-5	15°C	50% MA	*Vibrio splendidus* LGP32 strain LGP32	NR_074953	99.77
AA50RT01	KU647862	*Ascidia* sp. AM-5	RT	50% MA	*Vibrio splendidus* LGP32 strain LGP32	NR_074953	99.53

Table S2. continued from previous page

Isolate, identifier	Isolate, accession number	Source, host	Source, isolation temperature	Source, isolation medium	Closest relative, name	Closest relative, accession number	Closest relative, nucleotide identity [%]
AA50RT02	KU647863	*Ascidia* sp. AM-5	RT	50% MA	*Aliivibrio salmonicida* LFI1238 strain LFI1238	NR_074847	99.12
AAMA0401	KU647864	*Ascidia* sp. AM-5	4°C	MA	*Colwellia psychrerythraea* strain ATCC 27364	NR_037047	98.37
AAMA0402	KU647865	*Ascidia* sp. AM-5	4°C	MA	*Colwellia psychrerythraea* strain ATCC 27364	NR_037047	98.59
AAMA0403	KU647866	*Ascidia* sp. AM-5	4°C	MA	*Colwellia psychrerythraea* strain ATCC 27364	NR_037047	98.39
AAMA1001	KU647867	*Ascidia* sp. AM-5	10°C	MA	*Vibrio splendidus* LGP32 strain LGP32	NR_074953	99.77
AAMA1002	KU647868	*Ascidia* sp. AM-5	10°C	MA	*Arcobacter suis* strain F41	NR_116729	94.07
AAMA1004	KU647869	*Ascidia* sp. AM-5	10°C	MA	*Psychrosphaera saromensis* strain SA4-48	NR_113052	99.66
AAMA1005	KU647870	*Ascidia* sp. AM-5	10°C	MA	*Sinobacterium caligoides* strain SCSWE24	NR_109307	97.59
AAMA1006	KU647871	*Ascidia* sp. AM-5	10°C	MA	*Moritella dasanensis* strain ArB 0140	NR_044156	98.78
AAMA1501	KU647872	*Ascidia* sp. AM-5	15°C	MA	*Colwellia aestuarii* strain SMK-10	NR_043509	98.17
AAMA1502	sequence not submitted due to bad quality	*Ascidia* sp. AM-5	15°C	MA	*Thalassomonas agarivorans* strain TMA1	NR_043649	77.13
AAMA1503	KU647873	*Ascidia* sp. AM-5	15°C	MA	*Colwellia psychrerythraea* strain ATCC 27364	NR_037047	98.96
AAMART01	KU647874	*Ascidia* sp. AM-5	RT	MA	*Shewanella fidelis* strain KMM 3582	NR_025195	99.44
AAMART02	KU647875	*Ascidia* sp. AM-5	RT	MA	*Flammeovirga kamogawensis* strain YS10	NR_041438	98.55
AAMART03	KU647876	*Ascidia* sp. AM-5	RT	MA	*Tropicibacter litoreus* strain R37	NR_117647	98.08
AAMART04	KU647877	*Ascidia* sp. AM-5	RT	MA	*Vibrio splendidus* LGP32 strain LGP32	NR_074953	99.88
AS50RT01	KU647878	*Ascidiella aspersa* AS-1	RT	50% MA	*Microbulbifer arenaceous* strain RSBr-1	NR_114885	98.44
AS50RT02	KU647879	*Ascidiella aspersa* AS-1	RT	50% MA	*Photobacterium phosphoreum* strain NBRC 103031	NR_114184	99.78
ASMA1501	KU647880	*Ascidiella aspersa* AS-1	15°C	MA	*Arcobacter butzleri* ED-1	NR_074567	94.02
ASMA1502	KU647881	*Ascidiella aspersa* AS-1	15°C	MA	*Microbulbifer arenaceous* strain RSBr-1	NR_114885	98.44
ASMART01	KU647882	*Ascidiella aspersa* AS-1	RT	MA	*Microbulbifer variabilis* strain Ni-2088	NR_041021	98.39
ASMART02	KU647883	*Ascidiella aspersa* AS-1	RT	MA	*Shewanella surugensis* strain c959	NR_040950	98.69
AV501501	KU647884	*Ascidiella* sp. AV-10	15°C	50% MA	*Endozoicomonas elysicola* strain MKT110	NR_041264	95.53
AV501502	KU647885	*Ascidiella* sp. AV-10	15°C	50% MA	*Endozoicomonas elysicola* strain MKT110	NR_041264	97.99
AV501503	KU647886	*Ascidiella* sp. AV-10	15°C	50% MA	*Endozoicomonas elysicola* strain MKT110	NR_041264	97.98
AV50RT01	KU647887	*Ascidiella* sp. AV-10	RT	50% MA	*Endozoicomonas elysicola* strain MKT110	NR_041264	97.98
AV50RT02	KU647888	*Ascidiella* sp. AV-10	RT	50% MA	*Endozoicomonas elysicola* strain MKT110	NR_041264	97.87
AV50RT03	KU647889	*Ascidiella* sp. AV-10	RT	50% MA	*Endozoicomonas elysicola* strain MKT110	NR_041264	97.87
AV50RT04	KU647890	*Ascidiella* sp. AV-10	RT	50% MA	*Endozoicomonas elysicola* strain MKT110	NR_041264	97.99
AV50RT05	KU647891	*Ascidiella* sp. AV-10	RT	50% MA	*Endozoicomonas elysicola* strain MKT110	NR_041264	97.99

Table S2. continued from previous page

Isolate, identifier	Isolate, accession number	Source, host	Source, isolation temperature	Source, isolation medium	Closest relative, name	Closest relative, accession number	Closest relative, nucleotide identity [%]
AVMA0401	KU647892	*Ascidiella* sp. AV-10	4°C	MA	*Photobacterium phosphoreum* strain NBRC 103031	NR_114184	99.89
AVMA1001	KU647893	*Ascidiella* sp. AV-10	10°C	MA	*Photobacterium phosphoreum* strain NBRC 103031	NR_114184	99.78
AVMA1002	KU647894	*Ascidiella* sp. AV-10	10°C	MA	*Acinetobacter lwoffii* strain JCM 6840	NR_113346	99.77
AVMA1003	KU647895	*Ascidiella* sp. AV-10	10°C	MA	*Endozoicomonas elysicola* strain MKT110	NR_041264	97.99
AVMA1005	KU647896	*Ascidiella* sp. AV-10	10°C	MA	*Endozoicomonas elysicola* strain MKT110	NR_041264	97.98
AVMA1501	KU647897	*Ascidiella* sp. AV-10	15°C	MA	*Endozoicomonas elysicola* strain MKT110	NR_041264	97.87
AVMA1502	KU647898	*Ascidiella* sp. AV-10	15°C	MA	*Shewanella surugensis* strain c959	NR_040950	98.69
AVMA1503	KU647899	*Ascidiella* sp. AV-10	15°C	MA	*Shewanella surugensis* strain c959	NR_040950	98.68
AVMART01	KU647900	*Ascidiella* sp. AV-10	RT	MA	*Endozoicomonas elysicola* strain MKT110	NR_041264	97.98
AVMART02	KU647901	*Ascidiella* sp. AV-10	RT	MA	*Shewanella halifaxensis* strain HAW-EB4	NR_074822	99.89
AVMART03	KU647902	*Ascidiella* sp. AV-10	RT	MA	*Psychrobacillus psychrodurans* strain 68E3	NR_025409	98.9
AVMART04	KU647903	*Ascidiella* sp. AV-10	RT	MA	*Microbulbifer variabilis* strain Ni-2088	NR_041021	98.54
AVMART05	KT364255, KT364256, KT364257	*Ascidiella* sp. AV-10	RT	MA	*Endozoicomonas elysicola* strain MKT110	NR_041264	96.63-97.84*
