# Supplementary material for: Endozoicomonas Are Specific, Facultative Symbionts of Sea Squirts
Source: Front Microbiol. 2016 Jul 12;7:1042. doi: 10.3389/fmicb.2016.01042 (PMC4940369; doi:10.3389/fmicb.2016.01042)
Supplement: Supplementary file 3 [file Table3.PDF]

Table S3. Distribution and prevalence of Endozoicomonas in ascidians. Data from this study is highlighted with bold font face. -, no data

Ascidian host species	PCR-based survey	Culturing	Specific PCR	DGGE	Overall prevalence	Overall prevalence [%]	Sampling location (of host featuring *Endozoicomonas*)	Reference
Aplidium protectans	0/1	-	-	-	0/1	0%	-	Erwin et al., 2014
Aplidium sp.	0/1	-	-	-	0/1	0%	-	Erwin et al., 2014
***Ascidia* sp.**	**3/6**	**0/2**	**11/16**	**-**	**14/24**	**58%**	**Gullmarsfjorden, Sweden**	**This study**
Ascidia sp.	0/1	-	-	-	0/1	0%	-	Tianero et al., 2014
Ascidiella aspersa	**2/3**	**0/1**	**-**	**-**	**2/4**	**50%**	**Mediterranean, Spain; Gullmarsfjorden, Sweden**	**This study**
Ascidiella scabra	**4/4**	**1/1**	**7/7**	**-**	**12/12**	**100%**	**Gullmarsfjorden, Sweden**	**This study**
***Ascidiella* sp.**	**-**	**1/1**	**4/4**	-	**4/4(a)**	**100%**	**Gullmarsfjorden, Sweden**	**This study**
Botrylloides sp.	1/1	-	-	-	1/1	100%	Southern California, USA	Tianero et al., 2014
Botrylloides leachi (c)	Present	-	-	-	n.a.	n.a.	New Zealand	Cahill et al., 2016
Botrylloides violaceus	0/1	-	-	-	0/1	0%	-	Tait et al., 2007
Botryllus schlosseri	**1/1**	**-**	**6/6**	**-**	**6/6(b)**	**100%**	**Limfjorden, Denmark; Gullmarsfjorden, Sweden**	**This study**
Botryllus schlosseri	0/1	-	-	-	0/1	0%	-	Tait et al., 2007
Botryllus schlosseri (c)	Present	-	-	-	n.a.	n.a.	New Zealand	Cahill et al., 2016
Ciona intestinalis	**0/1**	**-**	**5/5**	**-**	**5/6**	**83%**	**Gullmarsfjorden, Sweden**	**This study**
Ciona intestinalis	7/7	-	-	-	7/7	100%	Cape Cod, USA; Southern California, USA; Fusaro Lake, Italy	Dishaw et al., 2014
Ciona robusta (c)	Present	-	-	-	n.a.	n.a.	New Zealand	Cahill et al., 2016
Ciona savignyi (c)	Present	-	-	-	n.a.	n.a.	New Zealand	Cahill et al., 2016
Clavelina arafurensis	0/1	-	-	-	0/1	0%	-	Erwin et al., 2014
Clavelina meridionalis	0/3	-	-	-	0/3	0%	-	Erwin et al., 2014
Cystodytes dellechiajei(c)	Present	-	-	-	n.a.	n.a.	Western Mediterranean, Spain	Martinez-Garcia, 2007
Cystodytes sp.	0/3	-	-	-	0/3	0%	-	Tianero et al., 2014
Diazona violacea	-	-	-	1/3	1/3	33%	Western Mediterranean, Spain	Martinez-Garcia, 2010
Didemnum cf. *albopunctatum*	0/1	-	-	-	0/1	0%	-	Erwin et al., 2014
Didemnum cf. *granulatum*	0/1	-	-	-	0/1	0%	-	Erwin et al., 2014
Didemnum fulgens	0/2	-	-	-	0/2	0%	-	Lopez-Legentil et al., 2015b
Didemnum multispirale	0/3	-	-	-	0/3	0%	-	Erwin et al., 2014
Didemnum sp.	4/10	-	-	-	4/10	40%	Southern California, USA; Papua New Guinea	Tianero et al., 2014
Didemnum sp.	-	1/1	-	-	1/1	100%	North coast of São Paulo state, Brazil	Menezes et al., 2010
Didemnum sp.	0/2	-	-	-	0/2	0%	-	Erwin et al., 2014
Didemnum sp.	0/1	-	-	-	0/1	0%	-	Tait et al., 2007

Table S3. continued from previous page

Ascidian host species	PCR-based survey	Culturing	Specific PCR	DGGE	Overall prevalence	Overall prevalence [%]	Sampling location (of host featuring *Endozoicomonas*)	Reference
Ecteinascidia diaphanis	0/1	-	-	-	0/1	0%	-	Erwin et al., 2014
Ecteinascidia turbinata	0/2	-	-	-	0/2	0%	-	Tianero et al., 2014
Eudistoma amplum	0/3	-	-	-	0/3	0%	-	Erwin et al., 2014
Eudistoma sp.	2/2	-	-	-	2/2	100%	Florida Keys, USA	Tianero et al., 2014
Leptoclinides madara	0/2	-	-	-	0/2	0%	-	Erwin et al., 2014
Lissoclinum badium	0/5	-	-	-	0/5	0%	-	Tianero et al., 2014
Lissoclinum badium	0/2	-	-	-	0/2	0%	-	Erwin et al., 2014
Lissoclinum bistratum	0/1	-	-	-	0/1	0%	-	Tianero et al., 2014
Lissoclinum cf. *caspulatum*	0/1	-	-	-	0/1	0%	-	Erwin et al., 2014
Lissoclinum patella	0/3	-	-	-	0/1	0%	-	Tianero et al., 2014
Lissoclinum patella	0/1	-	-	-	0/1	0%	-	Erwin et al., 2014
Molgula manhattensis	0/2	-	-	-	0/2	0%	-	Tait et al., 2007
Perophora aff. *modificata*	0/1	-	-	-	0/1	0%	-	Erwin et al., 2014
Phallusia arabica	0/3	-	-	-	0/3	0%	-	Erwin et al., 2014
Phallusia julinea	0/1	-	-	-	0/1	0%	-	Erwin et al., 2014
Phallusia philippinensis	1/1	-	-	-	1/1	100%	Great Barrier Reef, Australia	Erwin et al., 2014
Polycarpa argentata	0/1	-	-	-	0/1	0%	-	Erwin et al., 2014
Polycarpa aurata	0/3	-	-	-	0/3	0%	-	Erwin et al., 2014
Polycitor giganteus	0/1	-	-	-	0/1	0%	-	Erwin et al., 2014
Polyclinella azemai	-	-	-	1/2	1/2	50%	Western Mediterranean, Spain	Martinez-Garcia, 2010
Pseudodistoma crucigaster	0/6	-	-	-	0/6	0%		Lopez-Legentil et al., 2016
Pycnoclavella diminuta	1/3	-	-	-	1/3	33%	Great Barrier Reef, Australia	Erwin et al., 2014
Pycnoclavella sp.	0/2	-	-	-	0/2	0%	-	Erwin et al., 2014
Pyura sp.	0/1	-	-	-	0/1	0%		Tianero et al., 2014
Styela clava	**-**	**-**	**1/4**	**-**	**1/4**	**25%**	**Limfjorden, Denmark**	**This study**
Styela plicata	0/3	-	-	-	0/3	0%	-	Erwin et al., 2012
Styela sp.	0/1	-	-	-	0/1	0%	-	Tianero et al., 2014
Synoicum castellatum	0/3	-	-	-	0/3	0%	-	Erwin et al., 2014
Trididemnum sp.	0/2	-	-	-	0/2	0%	-	Tianero et al., 2014

(a) One specimen was screened both by culturing and specific PCR
(b) One specimen was screened both during PCR-based survey and by specific PCR
(c) Based on publication no prevalence data could be inferred.

Cahill PL, Fidler AE, Hopkins GA, Wood SA. 2016. Geographically conserved microbiomes of four temperate water tunicates. *Environ Microbiol Rep*: **In Print**

Dishaw, L.J., Flores-Torres, J., Lax, S., Gemayel, K., Leigh, B., Melillo, D. et al. (2014) The gut of geographically disparate Ciona intestinalis harbors a core microbiota. *PLoS One* **9**: e93386.

Erwin, P.M., Pineda, M.C., Webster, N., Turon, X., and López-Legentil, S. (2014) Down under the tunic: bacterial biodiversity hotspots and widespread ammonia-oxidizing archaea in coral reef ascidians. *ISME J* **8**: 575-588.

López-Legentil, S., Turon, X., and Erwin, P.M. (2016) Feeding cessation alters host morphology and bacterial communities in the ascidian Pseudodistoma crucigaster. *Front Zool* **13**: 1-11.

López-Legentil, S., Legentil, M.L., Erwin, P.M., and Turon, X. (2015a) Harbor networks as introduction gateways: contrasting distribution patterns of native and introduced ascidians. *Biol Invasions* **17**: 1623-1638.

López-Legentil, S., Turon, X., Espluga, R., and Erwin, P.M. (2015b) Temporal stability of bacterial symbionts in a temperate ascidian. *Front Microbiol* **6**: 1022.

Martínez-García, M., Díaz-Valdés, M., and Antón, J. (2010) Diversity of pufM genes, involved in aerobic anoxygenic photosynthesis, in the bacterial communities associated with colonial ascidians. *FEMS Microbiol Ecol* **71**: 387-398.

Martínez-García, M., Díaz-Valdés, M., Wanner, G., Ramos-Esplá, A., and Antón, J. (2007) Microbial community associated with the colonial ascidian Cystodytes dellechiajei. *Environ Microbiol* **9**: 521-534.

Menezes, C.B.A., Bonugli-Santos, R.C., Miqueletto, P.B., Passarini, M.R.Z., Silva, C.H.D., Justo, M.R. et al. (2010) Microbial diversity associated with algae, ascidians and sponges from the north coast of São Paulo state, Brazil. *Microbiol Res* **165**: 466-482.

Tait, E., Carman, M., and Sievert, S.M. (2007) Phylogenetic diversity of bacteria associated with ascidians in Eel Pond (Woods Hole, Massachusetts, USA). *J Exp Mar Biol Ecol* **342**: 138-146.

Tianero, M.D.B., Kwan, J.C., Wyche, T.P., Presson, A.P., Koch, M., Barrows, L.R. et al. (2014) Species specificity of symbiosis and secondary metabolism in ascidians. *ISME J* **9**: 615-628.
