# Supplementary material for: Endozoicomonas Are Specific, Facultative Symbionts of Sea Squirts
Source: Front Microbiol. 2016 Jul 12;7:1042. doi: 10.3389/fmicb.2016.01042 (PMC4940369; doi:10.3389/fmicb.2016.01042)
Supplement: Supplementary file 4 [file Table4.PDF]

Table S4. Overview of perfomred screenings per specimen. Listed are the accession numbers of the generated sequencing data.

Identity	Specimen identifier	COI	16S rRNA sequence [26F/1492R]	16S rRNA sequence [GM1F/1075R]	16S rRNA sequence [GM1F/ENDO-1240R]	Isolates
Ascidia species						
Ascidia sp.*	AM-1	-	KU648105, KU648106	-	-	-
Ascidia sp.	AM-2	KU647825	KU648107-KU648111	-	-	-
Ascidia sp.	AM-3	KU647826	-	-	KU648112-KU648119	-
Ascidia sp.	AM-4	KU647827	-	-	KU648120, KU648121	-
Ascidia sp.	AM-5	KU647828	-	-	KU648122-KU648129	KU647850-KU647877
Ascidia sp.	AA-1	-	KU648048-KU648051	-	-	-
Ascidia sp.*	AA-4	-	KU648062, KU648063	-	-	-
Ascidia sp.	AA-5	KU647820	-	-	no PCR product	-
Ascidia sp.	AA-6	-	-	-	no PCR product	-
Ascidia sp.	AA-8	-	-	-	no PCR product	-
Ascidia sp.	AA-9	-	-	-	KU648067-KU648073	-
Ascidia sp.	AV-1	-	KU648256-KU648261	-	-	-
Ascidia sp.	AV-11	-	-	-	KU648210-KU648217	KU647904-KU647930
Ascidia sp.	AV-12	KU647837	KU648223-KU648255	-	KU648218-KU648222	-
Ascidia sp.	AV-2	-	no PCR product	-	-	-
Ascidia sp.	AV-3	-	-	-	no PCR product	-
Ascidia sp.	AV-6	-	-	-	KU648262-KU648267	-
Ascidia sp.	AV-8	-	-	-	KU648268-KU648274	-
Ascidia sp.	AJ-2	-	-	-	KU648081-KU648088	-

Table S4. continued from previous page

Identity	Specimen identifier	COI	16S rRNA sequence [26F/1492R]	16S rRNA sequence [GM1F/1075R]	16S rRNA sequence [GM1F/ENDO-1240R]	Isolates
Ascidiella species						
Ascidiella sp.	AJ-1	KU647822	-	-	KU648074-KU648080	-
Ascidiella sp.	AJ-3	KU647823	-	-	KU648089-KU648096	-
Ascidiella sp.	AJ-4	KU647824	-	-	KU648097-KU648104	-
Ascidiella sp.	AV-10	KU647836	-	-	KU648202-KU648209	KU647884-KU647903; KT364255-KT364257
Ascidiella aspersa						
A. aspersa	AA-14	KF309594	-	KU647972-KU647993	-	-
A. aspersa	AA-15	KF309533	-	KU647994-KU648029	-	-
A. aspersa	AA-16	KF309568	-	KU648030-KU648047	-	-
A. aspersa	AS-1	KU647829	-	-	KU648130-KU648138	KU647878-KU647883
Ascidiella scabra						
A. scabra	AA-10	KU647816	-	-	KU647931-KU647938	-
A. scabra	AA-11	KU647817	-	-	KU647939, KU647940	-
A. scabra	AA-12	KU647818	KU647948-KU647963	-	KU647941-KU647947	-
A. scabra	AA-13	KU647819	-	-	KU647964-KU647971	-
A. scabra	AA-2	KU647834	KU648052-KU648057	-	-	-
A. scabra	AA-3	KU647835	KU648058-KU648061	-	-	-
A. scabra	AA-7	KU647821	-	-	KU648064-KU648066	-
A. scabra	AS-2	KU647830	-	-	KU648139-KU648145	KT364258-KT364260
A. scabra	AS-3	KU647831	-	-	KU648146-KU648152	-
A. scabra	AS-4	KU647832	KU648160-KU648193	-	KU648153-KU648159	-
A. scabra	AS-5	KU647833	-	-	KU648194-KU648201	-

Table S4. continued from previous page

Identity	Specimen identifier	COI	16S rRNA sequence [26F/1492R]	16S rRNA sequence [GM1F/1075R]	16S rRNA sequence [GM1F/ENDO-1240R]	Isolates
Botryllus schlosseri						
B. schlosseri	BS-1	KU647838	-	-	KU648275-KU648282	-
B. schlosseri	BS-2	KU647839	KU648288-KU648320	-	KU648283-KU648287	-
B. schlosseri	BS-3	KU647840	-	-	KU648321-KU648326	-
B. schlosseri	BS-4	KU647841	-	-	KU648327-KU648334	-
B. schlosseri	BS-5	KU647842	-	-	KU648335-KU648340	-
B. schlosseri	BS-6	KU647843	-	-	KU648341-KU648344	-
Ciona intestinalis						
*C. Intestinalis**	CI-1	-	no PCR product	-	-	-
*C. Intestinalis**	CI-2	-	KU648345	-	-	-
*C. Intestinalis**	CI-3	-	no PCR product	-	-	-
*C. Intestinalis**	CI-4	-	no PCR product	-	-	-
*C. Intestinalis**	CI-5	KU647844	-	-	PCR product not sequenced	-
*C. Intestinalis**	CI-6	KU647845	-	-	PCR product not sequenced	-
C. intestinalis	CI-7	KU647846	-	-	KU648346-KU648353	-
C. intestinalis	CI-8	KU647847	-	-	KU648354-KU648360	-
C. intestinalis	CI-9	KU647848	-	-	KU648361-KU648368	-
Styela clava						
S. clava	SC-1	-	-	-	no PCR product	-
S. clava	SC-2	-	-	-	no PCR product	-
S. clava	SC-3	-	-	-	no PCR product	-
S. clava	SC-4	KU647849	-	-	KU648374-KU648381	-
Water samples						
Water sample	D_water	n.a.	-	-	KU648369-KU648373	-
Water sample	W_18m	n.a.	-	-	PCR product unspecific	-
Water sample	W_30m	n.a.	-	-	KU648382, KU648383	-
Water sample	W_Oscar	n.a.	-	-	KU648384-KU648390	-

* Due to inability to obtain PCR product, low sampling depth of the resultant clone libraries, or not sequenced PCR bands these samples were disregarded for calculating the prevalence of *Endozoicomonas*
