# Supplementary material for: Endozoicomonas Are Specific, Facultative Symbionts of Sea Squirts
Source: Front Microbiol. 2016 Jul 12;7:1042. doi: 10.3389/fmicb.2016.01042 (PMC4940369; doi:10.3389/fmicb.2016.01042)
Supplement: Supplementary file 5 [file Table5.PDF]

Table S5. 16S rRNA gene sequence identities between the ascidian-specific *Endozoicomonas* sub-clade and described *Endozoicomonas* species. *Endozoicomonas gorgoniicola* does not have a published paralog-1 16S rRNA gene sequence and was therefore not included in the analysis.

	Range of sequence identities relative to ascidian-specific subclade [%]
Ascidian-specific subclade	96.8-100
E. elysicola	96.5-98.0
E. atrinae	96.6-98.1
E. montiporae	94.5-95.8
E. numazuensis	95.1-96.7
E. euniceicola	93.9-95.1
