# Supplementary material for: Endozoicomonas Are Specific, Facultative Symbionts of Sea Squirts
Source: Front Microbiol. 2016 Jul 12;7:1042. doi: 10.3389/fmicb.2016.01042 (PMC4940369; doi:10.3389/fmicb.2016.01042)
Supplement: Supplementary file 7 [file Image1.PDF]

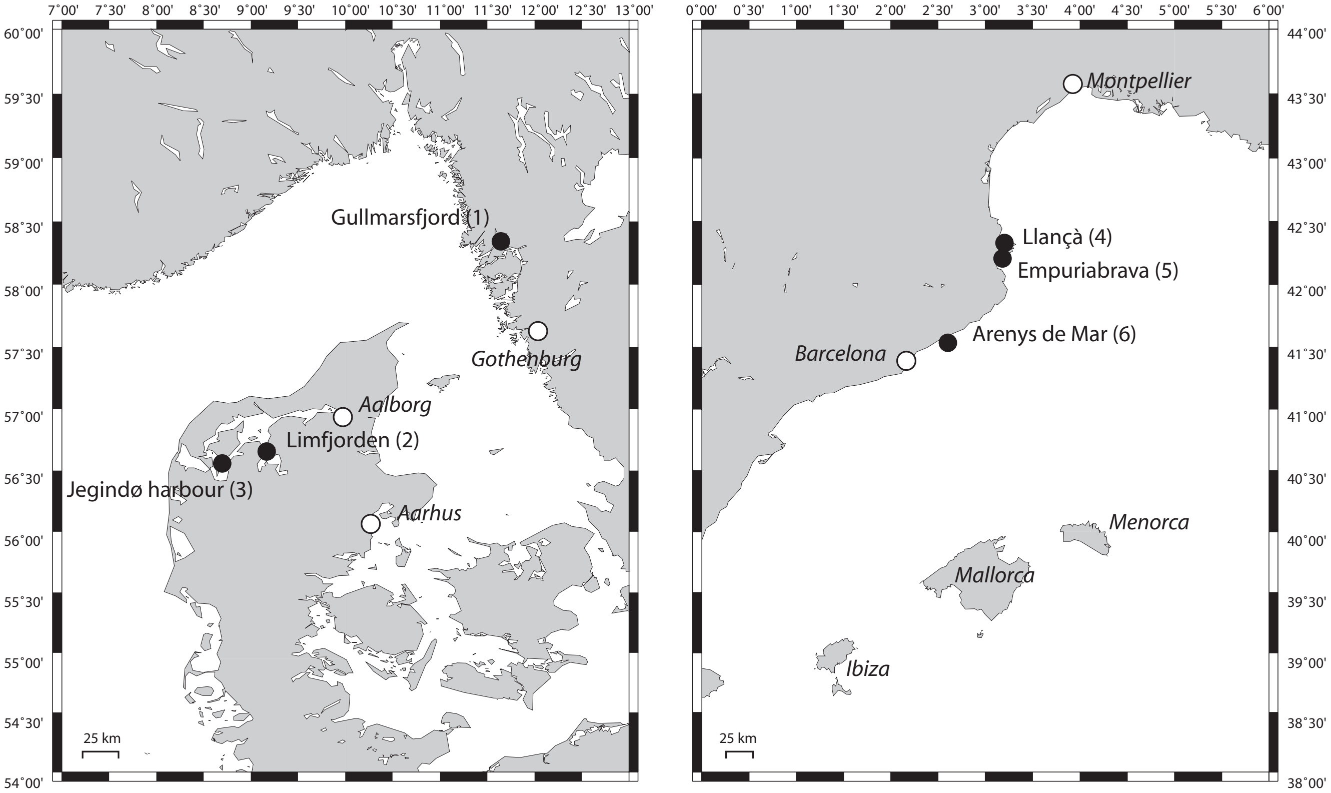

Figure S1. Sampling sites of ascidians examined in this study. Ascidian specimens were collected at the following sampling sites (black-filled dots): Gullmarsfjord at the west-coast of Sweden (1); Limfjorden (2) and the harbor of Jegindø, an island in the Limfjorden (3) both located in Northern Jutland, Denmark; and at Llançà (4), Empuriabrava (5) and Arenys de Mar (6) at the Mediterranean coast of Spain. Selected cities are shown for reference (open circles).
