# Supplementary material for: Endozoicomonas Are Specific, Facultative Symbionts of Sea Squirts
Source: Front Microbiol. 2016 Jul 12;7:1042. doi: 10.3389/fmicb.2016.01042 (PMC4940369; doi:10.3389/fmicb.2016.01042)
Supplement: Supplementary file 8 [file Image2.PDF]

A

```
References                                                     179                                                          242
Kistimonas asteriae (EU599216)                                 CCUACGGGGGAAAGCAGGGGA-UC----------UUCG--------G-ACCUUGCGCUAUUGGA
Endozoicomonas numazuensis (AB695089)                          CCUAAGGGGGAAAGCAGGGGA-UC----------UUCG--------G-ACCUUGCGCUACUGGA
Endozoicomonas elysicola (AB196667)                            CCUAAGGGGGAAAGGAGGGGACUC----------UUCG--------GAGCCUUUCGCUAUCAGA

Paralog 1
Endozoicomnas sp. AVMART05 (KT364256)                          CCUAAGGGGGAAAGGAGGGGA-UCGGC-------UUGC-------CG-ACCUUUCGCUAUCAGA
Endozoicomnas sp. KASP37 (KT364259)                            CCUAAGGGGGAAAGGAGGGGA-UCGGC-------UUGC-------CG-ACCUUUCGCUAUCAGA
Uncultured bacterium from Pocillopora damicornis (KC668735)    CCUAAGGGGGAAAGCAGGGGA-UCGG--------UUCG------CCG-ACCUUGCGCUAUCGGA

Paralog 2
Endozoicomnas sp. AVMART05 (KT364255)                          CCUAAGGGGGAAAGGAGGGGA-C--CCAUUUCUUUUAUUAGGAGUGA-GCCUUUCGCUAUCAGA
Endozoicomnas sp. KASP37 (KT364258)                            CCUAAGGGGGAAAGGAGGGGA-C--CCAUUUCUUUUAUUAGGAGUGA-GCCUUUCGCUAUCAGA
Uncultured bacterium from Pocillopora damicornis (KC668580)    CCUAAGGGGGAAAGCAGGGGC-C--CCG-UUCUUUUUUAAAGAGUGG-ACCUUGCGCUAUCGGA
```

B

```
References                                                     59                                       102
Kistimonas asteriae (EU599216)                                 AGCGGUAACAGAG-AGUG----CUUG------CACUCUGCUGAC
Endozoicomonas numazuensis (AB695089)                          AGCGGUAACAGAA-CUAG----CUUG------CUAGUUGCUGAC
Endozoicomnas elysicola (AB196667)                             AGCGGUAGCGGGA-AGAG----CUUG------CUCUUUGCCGAC

Putative paralog 1
Uncultured bacterium from Stylophora pistillata (KC669048)     AGCGGUAGCAGGA-AGUG----CUUG------CACUUCGCUGAC

Putative paralog 2
Uncultured bacterium from Stylophora pistillata (KC669050)     AGCGGUAGCGGGCCUGACCACUCUCGAGUGGUUCAGGCGCCGAC
```

Figure S2. Paralogs of the 16S rRNA gene detected in members of the *Endozoicomonas* clade. The shown numbering represents standard *E. coli* numbering of nucleotide positions. The only 16S rRNA gene type present in e.g. *Kistimonas asteriae*, *Endozoicomonas numazuensi*, and *Endozoicomonas elysicola* is shown for reference. (A) 16S rRNA gene paralogs detected in *Endozoicomonas* sp. AVMART05 and KASP37 (both isolated in the present study; Table S2), and in coral associated *Endozoicomonas*; e.g. representatives obtained from the coral species *Pocillopora damicornis*. The paralogs mainly differ in the hypervariable V2 region. (B) Putative second paralog featuring a divergent hypervariable V1 region and detected in representatives obtained from e.g. the coral species *Stylophora pistillata*.
