# Supplementary material for: Endozoicomonas Are Specific, Facultative Symbionts of Sea Squirts
Source: Front Microbiol. 2016 Jul 12;7:1042. doi: 10.3389/fmicb.2016.01042 (PMC4940369; doi:10.3389/fmicb.2016.01042)
Supplement: Supplementary file 9 [file Image3.PDF]

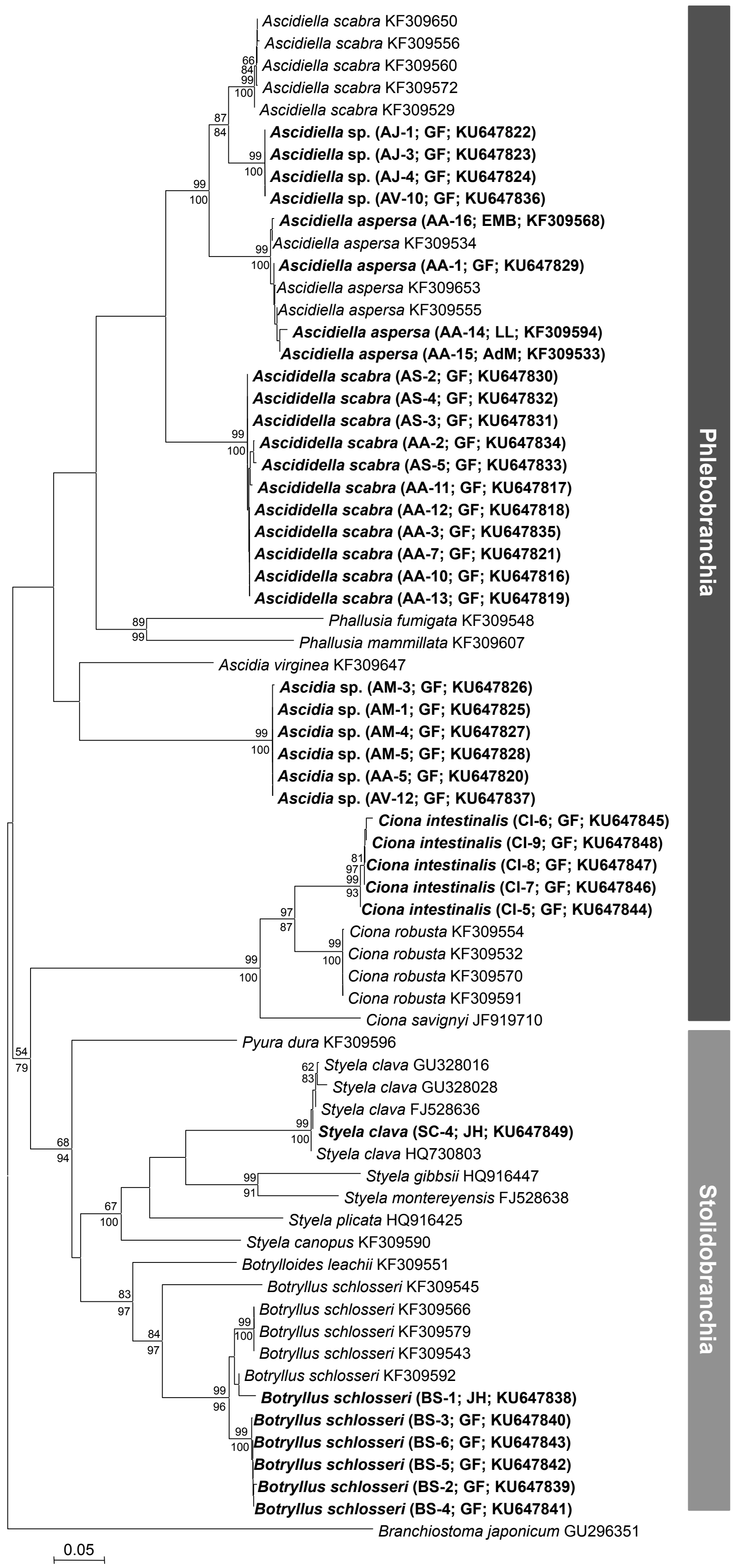

Figure S3. Phylogeny of ascidian hosts based on partial mitochondrial cytochrome c oxidase subunit I gene (CO1) sequences. Specimens analyzed in this study are highlighted in bold and include their identifier, origin, and accession number in parentheses. Labels on terminal nodes of reference sequences indicate the ascidian species and accession numbers. The tree topology was obtained from neighbor-joining (NJ) analysis. Bootstrap support percentages are located above (NJ) and below (maximum likelihood) the nodes (when ≥ 50% and retrieved for both phylogenetic analyses). Grey vertical bars indicate ascidian order. Scale bar represents 0.05 substitutions per site. Abbreviations: GF, Gullmarsfjord (Sweden); JH, Jegindø harbour (Limfjorden, Denmark); LL, Llan (Catalunya, Spain); EMB, Empuriabrava (Catalunya, Spain); AdM, Arenys de Mar (Catalunya, Spain).
