# Supplementary material for: Endozoicomonas Are Specific, Facultative Symbionts of Sea Squirts
Source: Front Microbiol. 2016 Jul 12;7:1042. doi: 10.3389/fmicb.2016.01042 (PMC4940369; doi:10.3389/fmicb.2016.01042)
Supplement: Supplementary file 11 [file Image5.PDF]

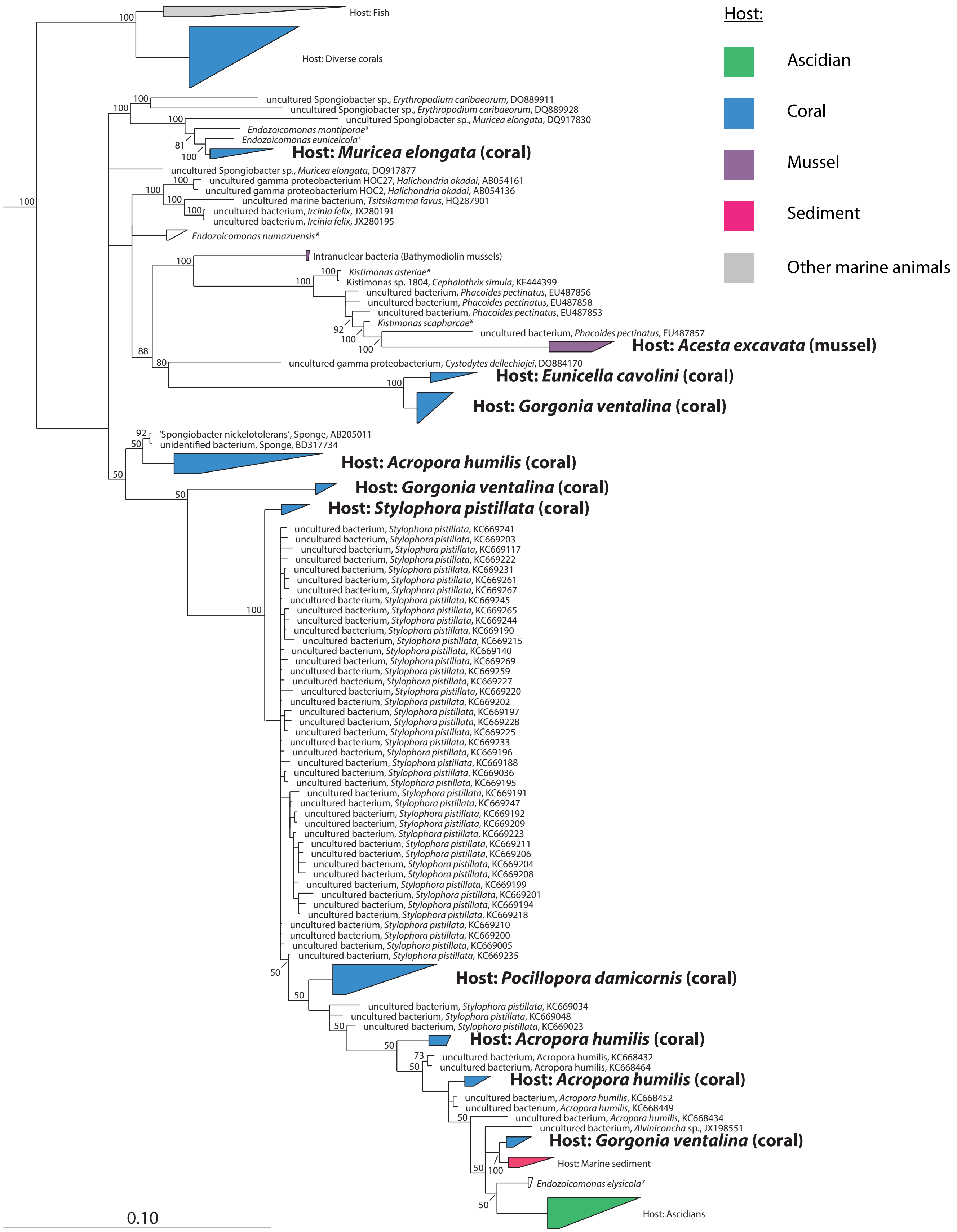

Figure S5. Phylogeny of the *Endozoicomonas* clade based on Bayesian inference. Individually shown sequences are followed by the corresponding host species and accession number. Described species of the *Endozoicomonas* clade are marked with an asterisk. Collapsed subclades are colour-coded by identity of the host material. Subclades containing sequences from a single host species are emphasized with increased font size and bold lettering. Sequences of the genera *Hahella* and *Zooshikella* were used as out-group (not shown). Numbers above or next to bifurcating nodes represent posterior probability support values. Scale bar represents 0.1 expected substitutions per site.
