# Supplementary material for: Endozoicomonas Are Specific, Facultative Symbionts of Sea Squirts
Source: Front Microbiol. 2016 Jul 12;7:1042. doi: 10.3389/fmicb.2016.01042 (PMC4940369; doi:10.3389/fmicb.2016.01042)
Supplement: Supplementary file 12 [file Image6.PDF]

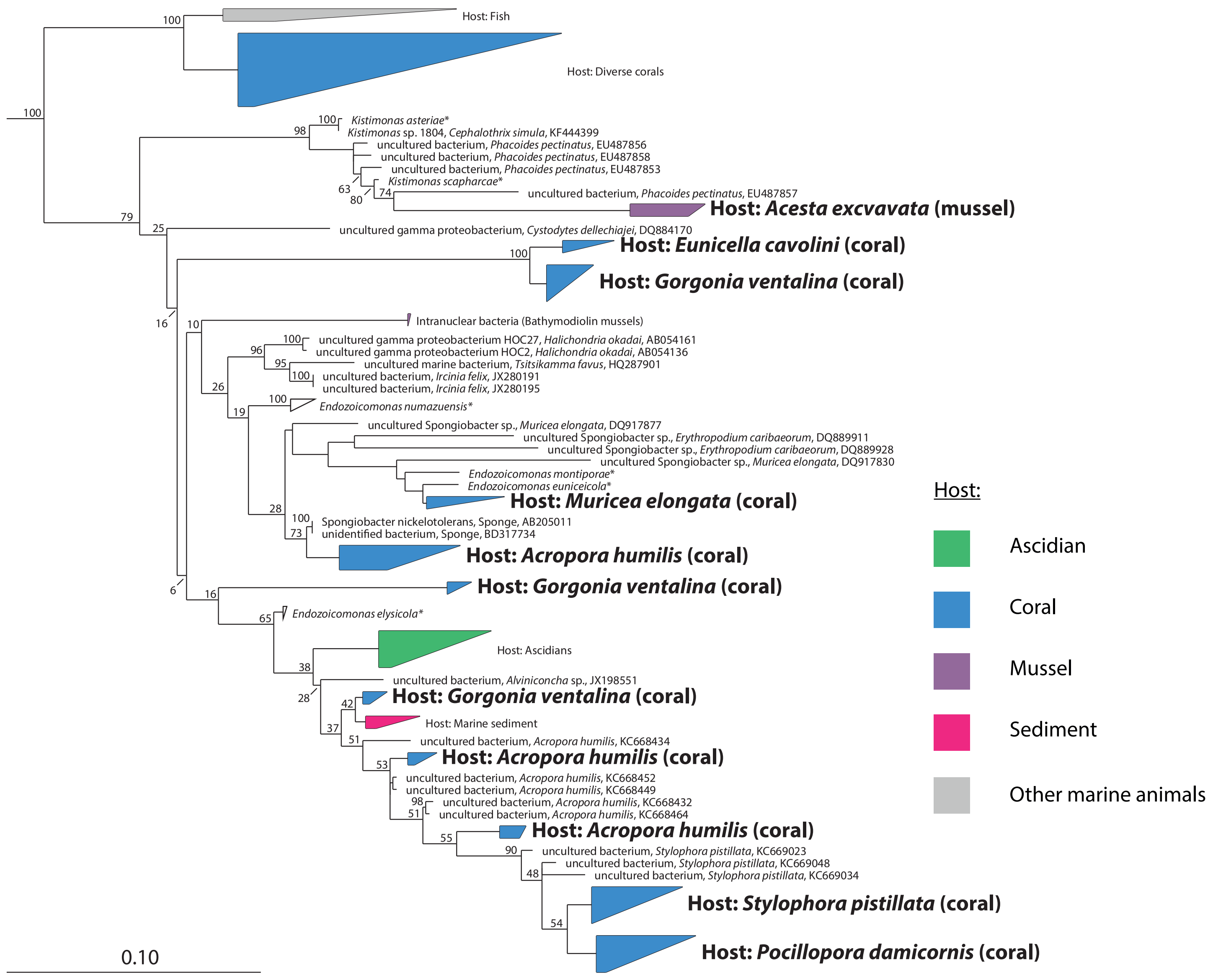

Figure S6. Phylogeny of the *Endozoicomonas* clade based on maximum likelihood analysis. The shown tree represents the consensus tree of 1,000 bootstrap analyses. Individually shown sequences are followed by the corresponding host species and accession number. Described species of the *Endozoicomonas* clade are marked with an asterisk. Collapsed subclades are colour-coded by identity of the host material. Subclades containing sequences from a single host species are emphasized with increased font size and bold lettering. Sequences of the genera *Hahella* and *Zooshikella* were used as outgroup (not shown). Numbers above and next to nodes represent boostrap support values [%]. Scale bar represents 0.1 expected substitutions per site.
