# Supplementary material for: Endozoicomonas Are Specific, Facultative Symbionts of Sea Squirts
Source: Front Microbiol. 2016 Jul 12;7:1042. doi: 10.3389/fmicb.2016.01042 (PMC4940369; doi:10.3389/fmicb.2016.01042)
Supplement: Supplementary file 13 [file Image7.PDF]

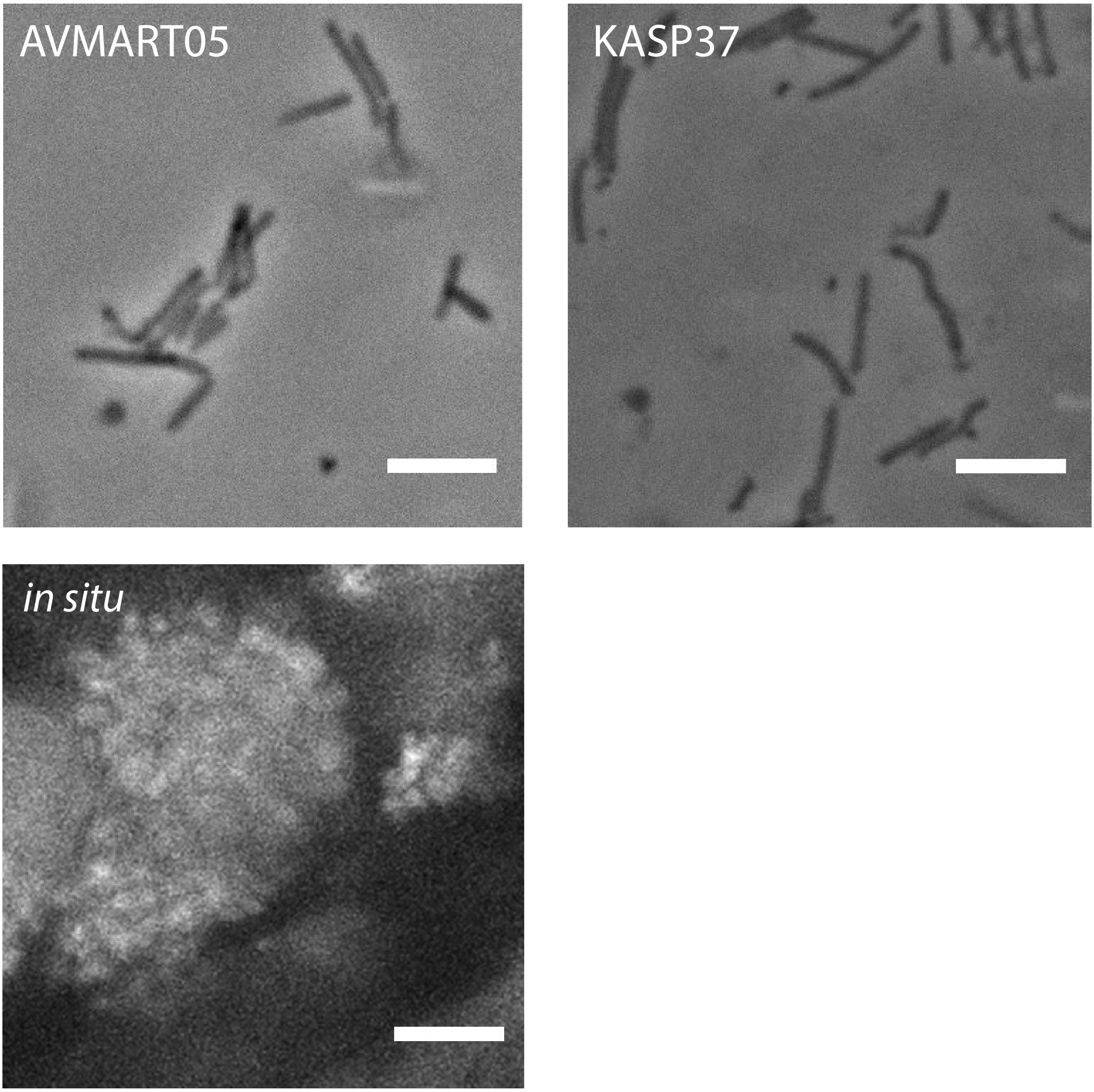

Figure S7. Cell morphology of *Endozoicomonas* isolates AVMART05 and KASP37 in comparison to *Endozoicomonas* morphology when associated with the pharynx of a *Ascidiella aspersa* specimen. *Endozoicomonas* isolates AVMART05 and KASP37 were visualized by phase-contrast microscopy. Pharynx-associated *Endozoicomonas* (labelled *in situ*) were visualized using the *Endozoicomonas*-specific probe ENDO-580. Scale bar represents 5 µm.
