# Supplementary material for: Endozoicomonas Are Specific, Facultative Symbionts of Sea Squirts
Source: Front Microbiol. 2016 Jul 12;7:1042. doi: 10.3389/fmicb.2016.01042 (PMC4940369; doi:10.3389/fmicb.2016.01042)
Supplement: Supplementary file 6 [file Table6.PDF]

Table S6. Classification of ascidian-derived *Endozoicomonas* sequences.

Identity	Specimen identifier	16S rRNA sequence [26F/1492R]				16S rRNA sequence [GM1F/1075R]			16S rRNA sequence [GM1F/ENDO-1240R]		
		Total generated sequences	Sequences classified as Endo-zoicomonas	Endo-zoicomonas sequences, paralog 1	Endo-zoicomonas sequences from ascidian subclade	Total generated sequences	Sequences classified as Endo-zoicomonas	Endo-zoicomonas sequences from ascidian subclade	Total generated sequences	Sequences classified as Endo-zoicomonas	Endo-zoicomonas sequences from ascidian subclade
Ascidia species											
Ascidia sp.	AM-1	2	0	-	-	-	-	-	-	-	-
Ascidia sp.	AM-2	5	1	1	1	-	-	-	-	-	-
Ascidia sp.	AM-3	-	-	-	-	-	-	-	8	7	7
Ascidia sp.	AM-4	-	-	-	-	-	-	-	2	2	2
Ascidia sp.	AM-5	-	-	-	-	-	-	-	8	8	1
Ascidia sp.	AA-1	4	0	-	-	-	-	-	-	-	-
Ascidia sp.	AA-4	2	0	-	-	-	-	-	-	-	-
Ascidia sp.	AA-5	-	-	-	-	-	-	-	-	-	-
Ascidia sp.	AA-6	-	-	-	-	-	-	-	-	-	-
Ascidia sp.	AA-8	-	-	-	-	-	-	-	-	-	-
Ascidia sp.	AA-9	-	-	-	-	-	-	-	7	7	7
Ascidia sp.	AV-1	6	4	4	4	-	-	-	-	-	-
Ascidia sp.	AV-11	-	-	-	-	-	-	-	8	8	8
Ascidia sp.	AV-12	33	1	1	0	-	-	-	6	5	0
Ascidia sp.	AV-2	-	-	-	-	-	-	-	-	-	-
Ascidia sp.	AV-3	-	-	-	-	-	-	-	-	-	-
Ascidia sp.	AV-4	-	-	-	-	-	-	-	-	-	-
Ascidia sp.	AV-5	-	-	-	-	-	-	-	-	-	-
Ascidia sp.	AV-6	-	-	-	-	-	-	-	6	6	6
Ascidia sp.	AV-7	-	-	-	-	-	-	-	-	-	-
Ascidia sp.	AV-8	-	-	-	-	-	-	-	7	7	5
Ascidia sp.	AV-9	-	-	-	-	-	-	-	-	-	-
Ascidia sp.	AJ-2	-	-	-	-	-	-	-	8	8	8

Table S6. continued from previous page

Identity	Specimen identifier	16S rRNA sequence [26F/1492R]				16S rRNA sequence [GM1F/1075R]			16S rRNA sequence [GM1F/ENDO-1240R]		
		Total generated sequences	Sequences classified as Endo-zoicomonas	Endo-zoicomonas sequences, paralog 1	Endo-zoicomonas sequences from ascidian subclade	Total generated sequences	Sequences classified as Endo-zoicomonas	Endo-zoicomonas sequences from ascidian subclade	Total generated sequences	Sequences classified as Endo-zoicomonas	Endo-zoicomonas sequences from ascidian subclade
Ascidiella species											
Ascidiella sp.	AJ-1	-	-	-	-	-	-	-	8	7	7
Ascidiella sp.	AJ-3	-	-	-	-	-	-	-	8	8	8
Ascidiella sp.	AJ-4	-	-	-	-	-	-	-	8	8	8
Ascidiella sp.	AV-10	-	-	-	-	-	-	-	8	8	7
Ascidiella aspersa											
A. aspersa	AA-14	-	-	-	-	22	2	2	-	-	-
A. aspersa	AA-15	-	-	-	-	36	33	10	-	-	-
A. aspersa	AA-16	-	-	-	-	18	0	-	-	-	-
A. aspersa	AS-1	-	-	-	-	-	-	-	9	9	9
Ascidiella scabra											
A. scabra	AA-7	-	-	-	-	-	-	-	3	3	3
A. scabra	AA-10	-	-	-	-	-	-	-	8	8	7
A. scabra	AA-11	-	-	-	-	-	-	-	2	2	2
A. scabra	AA-12	36	16	12	4	-	-	-	7	7	2
A. scabra	AA-13	-	-	-	-	-	-	-	8	8	5
A. scabra	AA-2	6	6	6	5	-	-	-	-	-	-
A. scabra	AA-3	4	4	4	4	-	-	-	-	-	-
A. scabra	AS-2	-	-	-	-	-	-	-	7	7	4
A. scabra	AS-3	-	-	-	-	-	-	-	7	7	1
A. scabra	AS-4	34	19	16	12	-	-	-	7	7	4
A. scabra	AS-5	-	-	-	-	-	-	-	8	8	5

Table S6. continued from previous page

Identity	Specimen identifier	16S rRNA sequence [26F/1492R]				16S rRNA sequence [GM1F/1075R]			16S rRNA sequence [GM1F/ENDO-1240R]		
		Total generated sequences	Sequences classified as Endo-zoicomonas	Endo-zoicomonas sequences, paralog 1	Endo-zoicomonas sequences from ascidian subclade	Total generated sequences	Sequences classified as Endo-zoicomonas	Endo-zoicomonas sequences from ascidian subclade	Total generated sequences	Sequences classified as Endo-zoicomonas	Endo-zoicomonas sequences from ascidian subclade
Botryllus schlosseri											
B. schlosseri	BS-1	-	-	-	-	-	-	-	8	8	0
B. schlosseri	BS-2	33	7	7	0	-	-	-	5	5	0
B. schlosseri	BS-3	-	-	-	-	-	-	-	6	6	0
B. schlosseri	BS-4	-	-	-	-	-	-	-	8	6	2
B. schlosseri	BS-5	-	-	-	-	-	-	-	8	1	1
B. schlosseri	BS-6	-	-	-	-	-	-	-	7	2	0
Ciona intestinalis											
C. intestinalis	CI-1	-	-	-	-	-	-	-	-	-	-
C. intestinalis	CI-2	1	0	-	-	-	-	-	-	-	-
C. intestinalis	CI-3	-	-	-	-	-	-	-	-	-	-
C. intestinalis	CI-4	-	-	-	-	-	-	-	-	-	-
C. intestinalis	CI-5	-	-	-	-	-	-	-	-	-	-
C. intestinalis	CI-6	-	-	-	-	-	-	-	-	-	-
C. intestinalis	CI-7	-	-	-	-	-	-	-	8	8	7
C. intestinalis	CI-8	-	-	-	-	-	-	-	8	7	7
C. intestinalis	CI-9	-	-	-	-	-	-	-	8	8	8
Styela clava											
S. clava	SC-1	-	-	-	-	-	-	-	-	-	-
S. clava	SC-2	-	-	-	-	-	-	-	-	-	-
S. clava	SC-3	-	-	-	-	-	-	-	-	-	-
S. clava	SC-4	-	-	-	-	-	-	-	8	8	8
Water samples											
Water sample	D_water	-	-	-	-	-	-	-	5	1	0
Water sample	W_18m	-	-	-	-	-	-	-	4	0	0
Water sample	W_30m	-	-	-	-	-	-	-	6	0	0
Water sample	W_Oscar	-	-	-	-	-	-	-	7	4	1
